# RIT1 Promotes the Proliferation of Gliomas Through the Regulation of the PI3K/AKT/c‐Myc Signalling Pathway

**DOI:** 10.1111/jcmm.70362

**Published:** 2025-01-20

**Authors:** Zhen Liu, Hao‐dong Jiang, Hao‐yuan Kan, Li Zhang, Yu‐xin Rao, Xiao‐bing Jiang, Ming‐hui Li, Qi Wang

**Affiliations:** ^1^ Department of Neurosurgery, Union Hospital, Tongji Medical College Huazhong University of Science and Technology Wuhan Hubei China; ^2^ Department of Anesthesiology Hubei University of Chinese Medicine Affiliated Hubei Hospital of Chinese Medicine Wuhan China; ^3^ Department of Anesthesiology, Union Hospital, Tongji Medical College Huazhong University of Science and Technology Wuhan China; ^4^ Institute of Anesthesia and Critical Care Medicine, Union Hospital, Tongji Medical College Huazhong University of Science and Technology Wuhan China; ^5^ Key Laboratory of Anesthesiology and Resuscitation, Huazhong University of Science and Technology, Ministry of Education Wuhan China

**Keywords:** gliomas, PI3K/AKT, proliferation, RIT1

## Abstract

Recently, RIT1 has been implicated in a range of neurological disorders; however, its precise function in glioma pathogenesis is not yet well‐defined. This study employed quantitative reverse transcription PCR (qRT‐PCR), Western blotting (WB), immunohistochemistry (IHC) and additional methodologies to assess RIT1 expression levels in glioma tissues. Furthermore, the study investigated its influence on glioma progression through a series of functional experiments. Animal models were also utilised to elucidate the mechanistic role of RIT1, with a particular focus on its effects on the PI3K/AKT signalling pathway. Research findings showcased that RIT1 is significantly overexpressed in gliomas and exhibits a strong correlation with tumour grade and unfavourable clinical outcomes. Furthermore, RIT1 serves as an independent prognostic marker of poor prognosis. Functional assays demonstrate that RIT1 facilitates the aggressiveness of glioma cells by activating the PI3K/AKT signalling. Additionally, it promotes tumour proliferation by inhibiting apoptosis and accelerating cell cycle progression. This study demonstrates that RIT1 significantly contributes to the aggressive phenotype and unfavourable prognosis of glioma, indicating its ability as a therapeutic target for glioma treatment.

## 1 Introduction

Glioma represents the predominant primary brain tumour, liable for 30% of all brain tumours and 80% of all malignant brain tumours, thereby considered the most common cause of death from primary brain tumours [1, 2, 3]. Among them, glioblastoma (GBM) constitutes the most malignant type, with less than 5% five‐year survival rate. In the current diagnostic categories of gliomas, there are usually some landmark molecular markers: 1p/19q coding deletion, mutation and MGMT promoter methylation, among others. Different molecular marker levels represent differences in the prognosis of different types of gliomas [4, 5]. At present, the main treatment plan for glioma is to carry out effective adjuvant treatments such as radiotherapy, chemotherapy and immunotherapy based on maximum safe resection, considering the pathological grade and molecular typing of glioma patients to improve patient survival as much as possible [6, 7]. However, the survival period of patients who have glioma, especially GBM, has not been significantly prolonged. Accordingly, deeply exploring new driving molecules in glioma occurrence and development, and developing targeted therapies is crucial to promote glioma therapeutic effect.

The RIT1 gene encodes a small GTP enzyme belonging to the Ras superfamily. RIT1 protein has a typical GTP‐binding domain and can exert its biological functions in a GTP‐binding state [8, 9]. Like other Ras family members, RIT1 contributes to cellular signal transduction and regulation of cell proliferation, differentiation and survival. Recently, the implication of the RIT1 gene in tumours has gradually attracted attention, as the occurred mutations have been found to exhibit a close correlation with cancer occurrence and progression, such as lung adenocarcinoma [10], hepatocellular carcinoma [11], endometrial carcinoma [12] and leukaemia [13, 14], among others [15]. Mutations in RIT1 mainly occur in the SwitchII domain of the RIT1 protein, usually altering the functionality of the RIT1 protein. This abnormally activates classic pathways such as MAPK and PI3K/AKT and promotes tumour proliferation and survival [16]. In some malignant tumours such as lung adenocarcinoma, RIT1 mutations appear to be mutually exclusive with mutations in currently known driver genes such as KRAS and EGFR, which suggests that RIT1 may be an independent oncogenic factor in some tumours [17]. Therefore, the role of RIT1 in cancer development remains controversial and requires specific analysis based on specific tissue types. In addition to its role in various solid tumours, RIT1's function in the nervous system has also gradually attracted attention. Currently, RIT1 has been found to be crucial in neuron morphogenesis, differentiation, survival and development [18]. However, there was no research yet has focused on the role of RIT1 in the development of glioma. The role of PI3K/AKT signalling has been reported in glioma development and chemotherapy resistance [19, 20], but whether RIT1 mediates its activation remains unknown.

Accordingly, we aimed to explore the role of RIT1 in glioma, explore new possible mechanisms that mediate glioma development, and provide new potential drug targets for subsequent treatment.

## 2 Materials and Methods

### 2.1 Bioinformatics Database

The glioma gene expression data were downloaded by accessing the TCGA database. The data was analysed employing cBioPortal as well as GraphPad Prism 7 Software (GraphPad Prism Software Inc., San Diego, California, USA). Regarding comprehensive solutions, kindly refer to the relevant publications mentioned here [21, 22, 23].

### 2.2 Cell Lines and Reagents

The NHA, U‐251, T98G and LN‐18 (Chinese National Infrastructure of Cell Line Resource, Beijing, China) and the HA, LN‐229, A‐172 and U‐87MG cell lines (American Type Culture Collection) were authenticated using short tandem repeat (STR) DNA fingerprinting before the commencement of studies. The cell lines were cultivated in DMEM (Hyclone, Logan County, KY, USA) that contained 10% fetal bovine serum (FBS; Gibco, Grand Island, NE, USA), 1% penicillin (100 U/L) and streptomycin (100 mg/L; both from Gibco) at 37°C in a 5% CO_2_ environment. Mycoplasma contamination was systematically assessed utilising the LookOut Mycoplasma PCR Detection Kit (Sigma‐Aldrich).

For further methodological details, please consult our published article [21, 24]. Trametinib (GSK1120212) was purchased from Selleck. cn. Table S3 provides details on all antibodies.

### 2.3 Patients

Tumour specimens were obtained from patients undergoing neurosurgical resection of gliomas. Immediately post‐resection, the specimens were cryopreserved at −80°C to maintain their molecular integrity for subsequent analyses. The collection process adhered to ethical guidelines and regulations, and all patients signed informed consent before the surgical operations, permitting both the intervention and the anonymized scientific analysis of their diseased tissue. The consent forms were created to advise participants that their tissue samples were going to be used for scientific research while preserving the confidentiality of their personal data. The Human Research Committee of Wuhan union hospital and the China Anti‐Cancer Association (CACA) authorised the guidelines and rules for the collection and use of human tissue samples in research projects. These regulatory bodies are tasked with supervising ethical considerations and safeguarding patients' rights and welfare during the study procedure.

### 2.4 Plasmid Constructs and Transfection

Lentiviruses that encode short hairpin RNAs (shRNAs) specific to the RIT1 and c‐Myc genes were procured from Shandong ViGene, a biotechnology firm located in Shandong, China. Lentiviruses were utilised to transduce cells, enabling the suppression of RIT1 and c‐Myc gene expression. Table S4 lists the used shRNA sequences. The engineered shRNAs preferentially target the mRNA of RIT1 and c‐Myc, promoting their breakdown and so reducing their production in the infected cells. Control experiments involved infecting cells with either an empty vector or scrambled shRNA sequences. The control groups are crucial for assessing the specificity and efficiency of gene knockdown by facilitating comparisons with cells infected with RIT1 and c‐Myc‐targeting shRNAs.

For further detailed methodologies and results, please consult the relevant documents provided [25, 26].

### 2.5 Cell Migration and Invasion Assays

A Transwell chamber with a suitable pore size was selected according to the experimental requirements. For invasion experiments, matrix gel (Matrigel) was applied to the bottom of the Transwell chamber to simulate the extracellular matrix. The treated tumour cells were suspended in serum‐free culture media and inoculated onto the chamber's upper layer. The typical cell inoculation per well was 1 × 10^4^–1 × 10^5^. A complete growth medium with 10% FBS was introduced into the lower compartment of the Transwell chamber and subjected to incubation for a specified duration. After incubation, the Transwell chamber was extracted and rinsed with phosphate‐buffered saline (PBS), and the non‐migrated/invaded cells were delicately eliminated from the upper layer. Meanwhile, we fixed and stained the migrated/invaded cells at the bottom of the chamber with crystal violet. To evaluate the migration/invasion ability of the cells, the number of passed cells through the membrane was observed and counted under a microscope.

### 2.6 Luciferase Gene Reporter Assay

Aiming at constructing a test plasmid, a reporter plasmid containing dual luciferase genes was selected, followed by inserting the target gene upstream of the luciferase gene. At the same time, a plasmid with an internal reference gene was used as a control to standardise the data. Before the experiment, a suitable cell line was cultured to a suitable density, and both the constructed reporter plasmid and internal reference plasmid were transfected into the cells, thereby improving the transfection efficiency with an appropriate transfection reagent (such as liposome). According to experimental needs, the transfected cells were specifically treated to detect the activity of the target regulatory sequence under different conditions. After treatment, the collected cells were lysed by a lysis buffer to release intracellular luciferase protein. The luciferase activity of each group of samples was measured via a luciferase activity detector using a luciferase detection reagent by first reading the internal reference luciferase activity, followed by the target luciferase activity. The obtained luciferase data were normalised (the target luciferase activity value was divided by the internal reference activity value), and the relative expression levels of luciferase under different experimental conditions were analysed to evaluate the activity changes of the target regulatory sequence.

### 2.7 Cell Counting Kit‐8 Assay

Upon entering the logarithmic growth phase, characterised by vigorous cell division, the used cells were resuspended and seeded onto 96‐well plates (5 × 10^3^ cells/well). The utilised culture medium was deemed inadequate, indicating the lack of specific additions or components required for experimental circumstances. After seeding, the cells were allowed a 24‐h period to adhere to the well surface, promoting their adhesion and the establishment of a monolayer. Subsequently, the culture media was discarded, and the cells were treated with cell counting kit‐8 (CCK‐8) (10 μL) as per the manufacturer's guidelines. The CCK‐8 is a commonly employed reagent in tests that evaluate cell viability and proliferation, as it measures the metabolic activity of cells, thereby indicating their viability. Absorbance measurements were performed utilising a microplate reader calibrated to 450 nm. This wavelength is commonly utilised to identify the signal produced by CCK‐8, which signifies cell viability. The baseline reading, indicative of the background absorbance of the medium or reagents, was subtracted from the observed absorbance values to assure precision.

### 2.8 Colony Formation Assay

The cells in different treatment groups were appropriately diluted, and about 500 cells were inoculated into each well to ensure that single cells could form independent clones. The cells were then inoculated into 6‐well plates a 37°C in a 5% CO_2_ incubator for about 2 weeks until visible clones were formed. During the culture period, we regularly changed the culture medium to maintain adequate nutrition and remove metabolic waste. Thereafter, the cells were subjected to 4% paraformaldehyde fixation for 15–20 min and crystal violet staining for 30 min to make the colonies clearly visible. The stained culture dish was rinsed with water to remove excess dye, the number of colonies formed was counted (clusters containing at least 50 cells are usually considered valid clones), and the clone formation rate was calculated.

### 2.9 Western Blotting

The collected cell or tissue samples were lysed with lysis buffer, and total protein was extracted. Protein concentration was determined through the BCA method to ensure that the amount of protein in each group of samples was consistent. The protein sample was mixed with loading buffer and heat‐denatured, loaded onto an SDS‐PAGE gel, and electrophoresed to separate the proteins according to their molecular weight. The proteins in the gel were then transferred to a PVDF or nitrocellulose membrane. Then, a blocking solution containing 5% skim milk powder or bovine serum albumin (BSA) was deployed to block nonspecific binding sites on the membrane, usually for 1 h, to reduce nonspecific background signals. Finally, the membrane was placed in a solution containing the primary antibody and incubated for a whole night at 4°C to ensure that the antibody specifically binds to the target protein. After washing away the unbound primary antibody, the HRP‐labelled secondary antibody (antibody against the primary antibody) was introduced and incubated for 1 h at room temperature to further amplify the signal. The ECL chemiluminescent reagent was utilised for colour development, the target protein signal was detected on the membrane via a chemiluminescent instrument, and the band intensity was recorded.

### 2.10 Immunohistochemical and Immunofluorescence Staining Analyses

The methodology for immunofluorescence (IF) staining and IHC, along with the evaluation scoring system, is delineated as follows:

For IF staining, tissue sections were fixed in 4% paraformaldehyde to preserve tissue architecture and maintain protein integrity. Thereafter, the sections were permeabilized using 0.5% Triton X‐100 to enable antibody infiltration into the cellular structures. Tissue sections were subsequently washed three times with PBS to remove any remaining fixative or permeabilizing agent, followed by treatment with a blocking buffer that contained 5% BSA to prevent nonspecific antibody binding. Target‐specific antibodies were suspended in a blocking buffer and treated with the tissue sections overnight at an optimal temperature. These primary antibodies adhered to their corresponding target proteins, enabling their identification. The tissue sections were treated with secondary antibodies conjugated to Cy3, a fluorescent dye or another appropriate fluorophore. These secondary antibodies were engineered to precisely attach to primary antibodies, thus producing fluorescence signals.

The tissue sections underwent counterstaining with DAPI, a fluorescent stain that selectively binds to DNA, thereby facilitating the visualisation of cell nuclei. Subsequently, the sections were mounted with an anti‐fade solution to ensure the preservation of fluorescence signals. Finally, laser scanning confocal microscopy was used to observe the fluorescence signal, taking the Olympus FV500 system as an example, which can capture high‐resolution images of stained tissue sections. For IHC, formalin‐fixed, paraffin‐embedded tissue slices with a thickness of 4 μm were produced. The tissue sections were subsequently treated with a primary antibody specific to the target protein, such as RIT1. The tissue slices were treated with a Cy3‐conjugated secondary antibody specific to the primary antibody. This study utilised the semiquantitative scoring technique immunoreactive score (IRS), which is calculated by multiplying the staining intensity (SI) by the percentage of positive cells (PP%). The SI is classified as 0, 1, 2, 3 and 4, which denotes negative, mild, moderate, strong and very strong staining, respectively. The PP was delineated based on PPs%: 0 indicates less than 1%, 1 signifies 1%–10%, 2 denotes 11%–50%, 3 represents 51%–80%, and 4 exceeds 80% PPs. The multiplication of these two components produces the IRS, with potential scores varying from 0 to 16. Ultimately, 10 fields from various regions of each sample were assessed using the IRS, a scoring system that evaluates protein expression levels in tissue sections by analysing both the intensity as well as distribution of staining. Table S3 summarises the antibodies.

### 2.11 Cell Cycle Experiments

The cells were cultured to be tested to an appropriate density, collected and washed twice with PBS. Subsequently, 70% ice‐cold ethanol was gradually added to the suspended cells to minimise aggregation, followed by fixation at −20°C for 3 h. After fixation, the cells were centrifuged to eliminate the ethanol and subsequently rinsed with PBS to eradicate any remaining ethanol. Then, PBS solution containing RNase was introduced and incubated at 37°C for 30 min to digest the RNA to ensure that the stain only binds to DNA. Propidium iodide (PI) staining solution was added and incubated for 30 min at room temperature in a light‐proof environment. Finally, we employed flow cytometry to detect the stained cells, conducting data analysis via FlowJo software to segment the cell cycle distribution, which was usually divided into G0/G1, S and G2/M phases, and cell proportion in each cell cycle phase was evaluated.

### 2.12 Animal Experiments

Six‐ to eight‐week‐old female nude mice (Beijing Vital River Laboratory Animal Technology Co. Ltd.) were acclimatised in a mouse‐specific pathogen‐free (SPF) facility for 1 week prior to injection. For each independent experiment, 3–5 mice per cohort were utilised. Glioma cells from different treatment groups, with a number of approximately 5 × 10^5^, were inoculated orthotopically into the mouse skull. The inoculation site was located 1 mm in front of the bregma, 2 mm to the right, and 3 mm deep, with an inoculation volume of 5 μL. A month after inoculation, the mice were euthanized, and specimens were gathered for evaluation of tumour volume, H&E staining and Ki‐67 IF staining.

### 2.13 Statistical Analysis

Statistical analyses were conducted through SPSS software, version 25.0 (SPSS Inc., Chicago, IL, USA). Comparative analysis between two datasets included both unpaired and paired *t*‐tests while employing one‐way ANOVA for comparisons involving more than three datasets. The Shapiro–Wilk test, utilising an alpha level of 0.05, was conducted to evaluate the normality of the data distribution. Results are expressed as mean ± standard deviation (SD) or median with interquartile range (IQR). To compare the two groups, we used a *t*‐test, a corrected *t*‐test (*t*'), or a rank‐sum test, contingent upon the assumptions of normality and homogeneity of variance. Overall means for comparisons among different groups were assessed employing one‐way ANOVA, the Brown–Forsythe ANOVA test, or the rank‐sum test. To regulate the overall Type I error rate, corrections for pairwise comparisons were implemented utilising the Dunnett T3 test or Dunn's method for *p*‐value correction. A two‐sided *p*‐value of 0.05 indicated statistical significance.

## 3 Results

### 3.1 RIT1 Is Overexpressed in Glioma and Is Inversely Correlated With Prognosis

**FIGURE 1:**
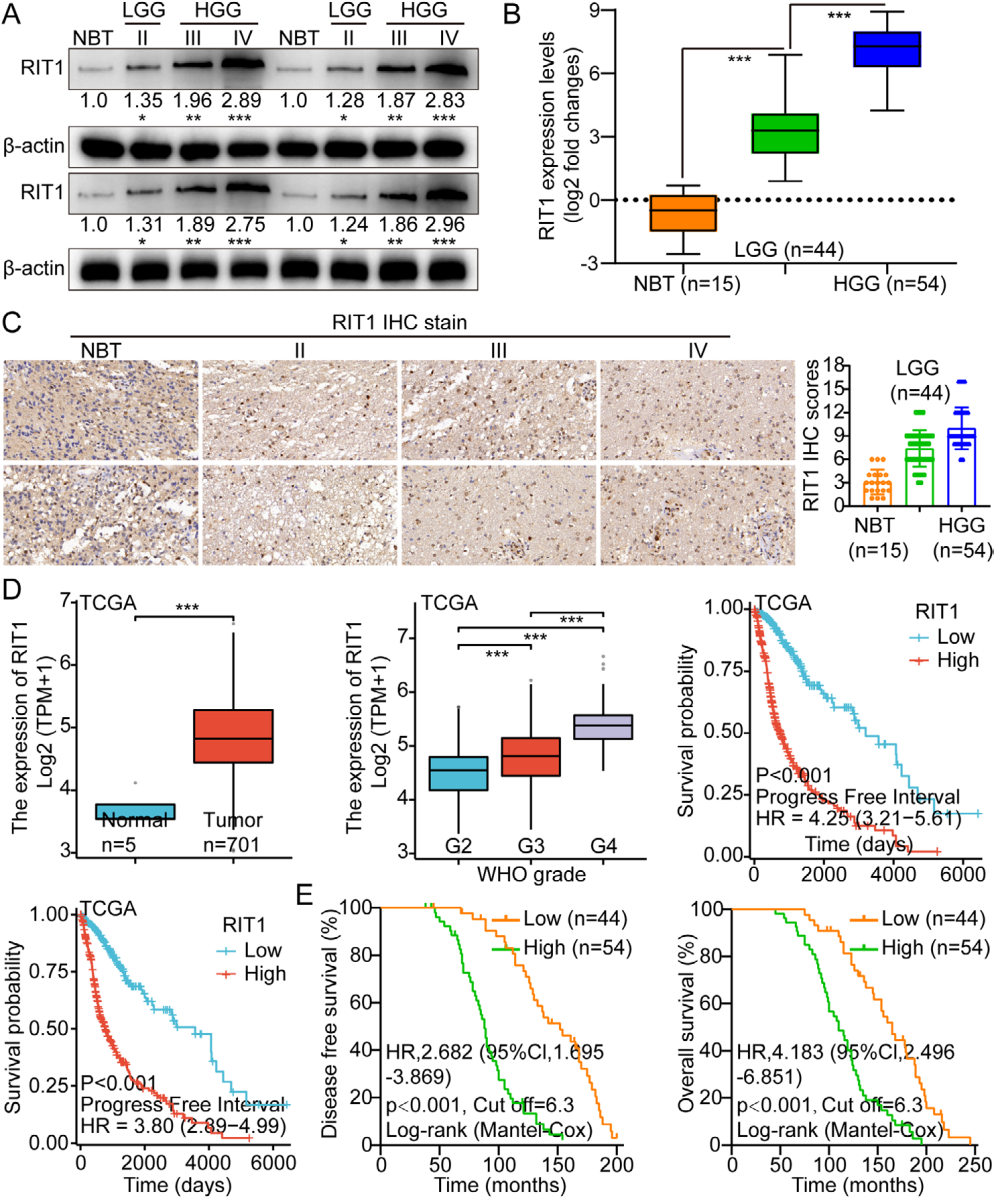
RIT1 is highly expressed in glioma and is inversely correlated with prognosis. (A) WB was employed to measure RIT1 expression in normal brain tissue and various glioma grades (LGG II: Low‐grade; HGG III + IV: High‐grade). (B) qRT‐PCR was employed to measure RIT1 mRNA levels in normal brain tissue and various grades of gliomas. (C) IHC staining and scoring measured RIT1 protein levels in normal brain tissue and various grades of gliomas. (D) The results of the TCGA database showed that RIT1 increased with the increase of tumour grade, and the expression level was inversely proportional to the patient's prognosis. (E) The results of clinical samples also showed that its expression level was inversely proportional to the patient's prognosis. Data are presented as mean ± SD from three independent experiments. *p < 0.05, **p < 0.01 and ***p < 0.001.

The research utilised the glioma entire gene expression map database from the TCGA, providing genomic sequence data for five normal brain tissue (NBT) samples and 701 glioma tissue samples. Unlike NBTs, RIT1 was significantly overexpressed in glioma tissues (Figure S1A), suggesting RIT1's potential involvement in glioma progression. The RT‐PCR was carried out foe additional investigation of RIT1 expression across different grades of human gliomas. The results demonstrated that RIT1 expression levels varied among different glioma grades (Figure S1A). Furthermore, WB analysis was performed on different cohorts of glioma tissue samples, encompassing NBTs as well as gliomas classified as grades II, III and IV. The results from the WB analysis demonstrated that glioma tissues exhibited elevated expression levels of RIT1 compared to NBTs. In addition, higher glioma grades were positively related to RIT1 overexpression at the RNA and protein levels (Figure 1A,B). The IHC results demonstrated a significant variation in the immune staining index (SI) of RIT1 across different glioma grades. Subsequent quantitative analyses corroborated the observed elevation in RIT1 protein expression (Figure 1C). Furthermore, K‐M analysis manifested that overexpressed RIT1 was related to reduced survival duration (Figure 1D,E). This suggests the potential utility of RIT1 as a prognostic glioma biomarker.

Herein, we performed a receiver operating characteristic (ROC) analysis to ascertain the predictive efficacy of RIT1‐based and WHO grade‐based models, as well as their combined application, in determining the pathological and clinical outcomes associated with RIT1. The findings revealed that the combined model (AUC = 0.712) demonstrated enhanced predictive capability for clinical outcomes compared to the WHO grade‐based model alone (AUC = 0.569, Figure S1B,C).

Furthermore, the examination of the association between RIT1 mRNA expression levels as well as clinicopathological features in a cohort of 98 patients with glioma (Table S1) revealed a significant interplay with tumour size (*p* < 0.001), Karnofsky Performance Status (KPS) (*p* = 0.012), WHO tumour stage (*p* < 0.001), and tumour recurrence (*p* = 0.025). Both univariate and multivariate Cox regression studies demonstrated that RIT1 mRNA expression correlated with the WHO stage and was recognised as an independent predictor of worse survival outcomes in glioma patients (Table S2). The data indicate that RIT1 could function as a prospective biomarker for glioma.

### 3.2 RIT1 Overexpression Enhances Glioma Cell Growth, Migration and Invasion

**FIGURE 2:**
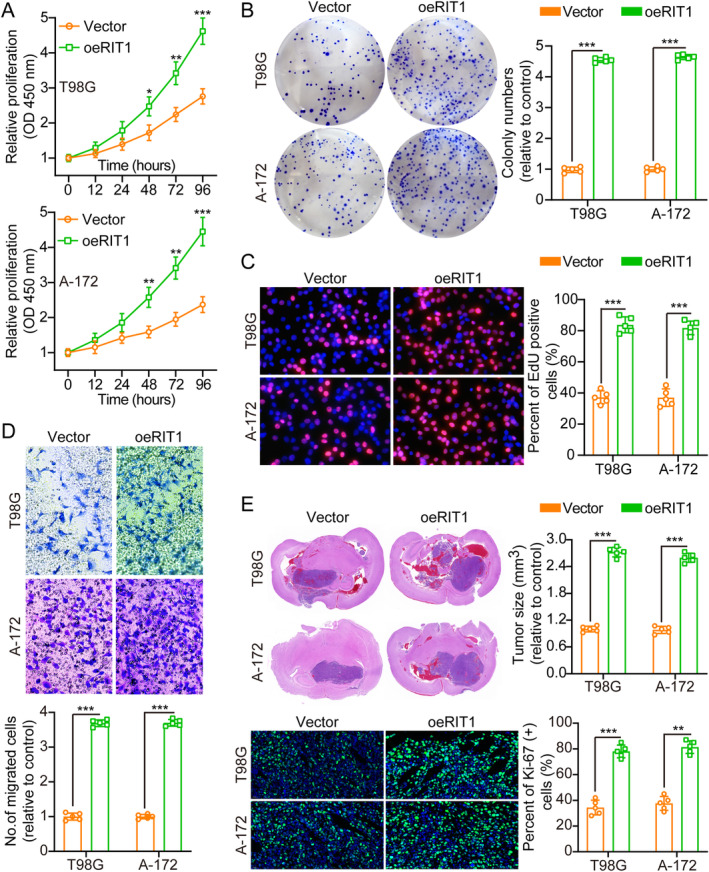
RIT1 overexpression enhances glioma cell growth, migration, and invasion. (A) CCK‐8 measured cell growth curves for Vector and oeRIT1, *n* = 5. (B) RIT1 overexpression enhanced colony formation and histogram analysis, *n* = 5. (C) EdU incorporation assays also indicated that RIT1 overexpression increased EdU‐positive cells, inferring increased proliferation of cells, *n* = 5. (D) Transwell migration assays indicate that RIT1 overexpression enhances cell migration, *n* = 5. Scale bars: 50 μm. (E) Representative pictures and histogram of tumour size and Ki‐67 staining for Vector and oeRIT1, *n* = 5. Data are presented as mean ± SD from five independent experiments. *p < 0.05, **p < 0.01 and ***p < 0.001.

Initially, RIT1 protein levels were measured in Normal HA, NHA and six glioma cell lines (U‐251, T98G, LN‐229, A‐172, LN‐18 and U‐87MG) using WB, revealing higher levels in glioma cells compared to HA and NHA (Figure S1D). Consequently, T98G and A‐172, with moderate RIT1 expression, were chosen for further study. Overexpression and knockdown of RIT1 between T98G and A‐172 cells were confirmed by WB (Figure S1E). Aiming at further clarifying the RIT1 effect on tumour cell proliferation, we used CCK8, clone formation assay, TUNEL and EdU assay to verify. All outcomes manifested that RIT1 overexpression could enhance tumour cell proliferation (Figure 2A–C). Furthermore, the transwell assay results elucidated that RIT1 overexpression significantly augmented glioma cell invasion and migration (Figure 2D and Figure S1F). This indicates that RIT1 may augment the migratory and invasive properties of glioma cells, a defining trait of malignant tumours.

To further investigate the influence of RIT1, annexin V staining and PI staining were conducted. Annexin V staining is a widely utilised method for the detection of apoptotic cells, whereas PI staining is employed to determine cell cycle status. The results revealed that RIT1 overexpression resulted in a reduction in cell apoptosis, suggesting a potential anti‐apoptotic effect. Additionally, oeRIT1 was observed to promote cell cycle progression (Figure S2A). Moreover, oeRIT1 facilitated tumour growth in vivo. The proliferative tumour index was evaluated using Ki‐67 IF staining, revealing significantly higher oeRIT1 group proliferation than in the control (Figure 2E). Collectively, RIT1 overexpression significantly diminishes cellular apoptosis and facilitates tumour progression in vitro and in vivo.

### 3.3 RIT1 Knockdown Inhibits Glioma Cell Growth, Migration and Invasion

**FIGURE 3:**
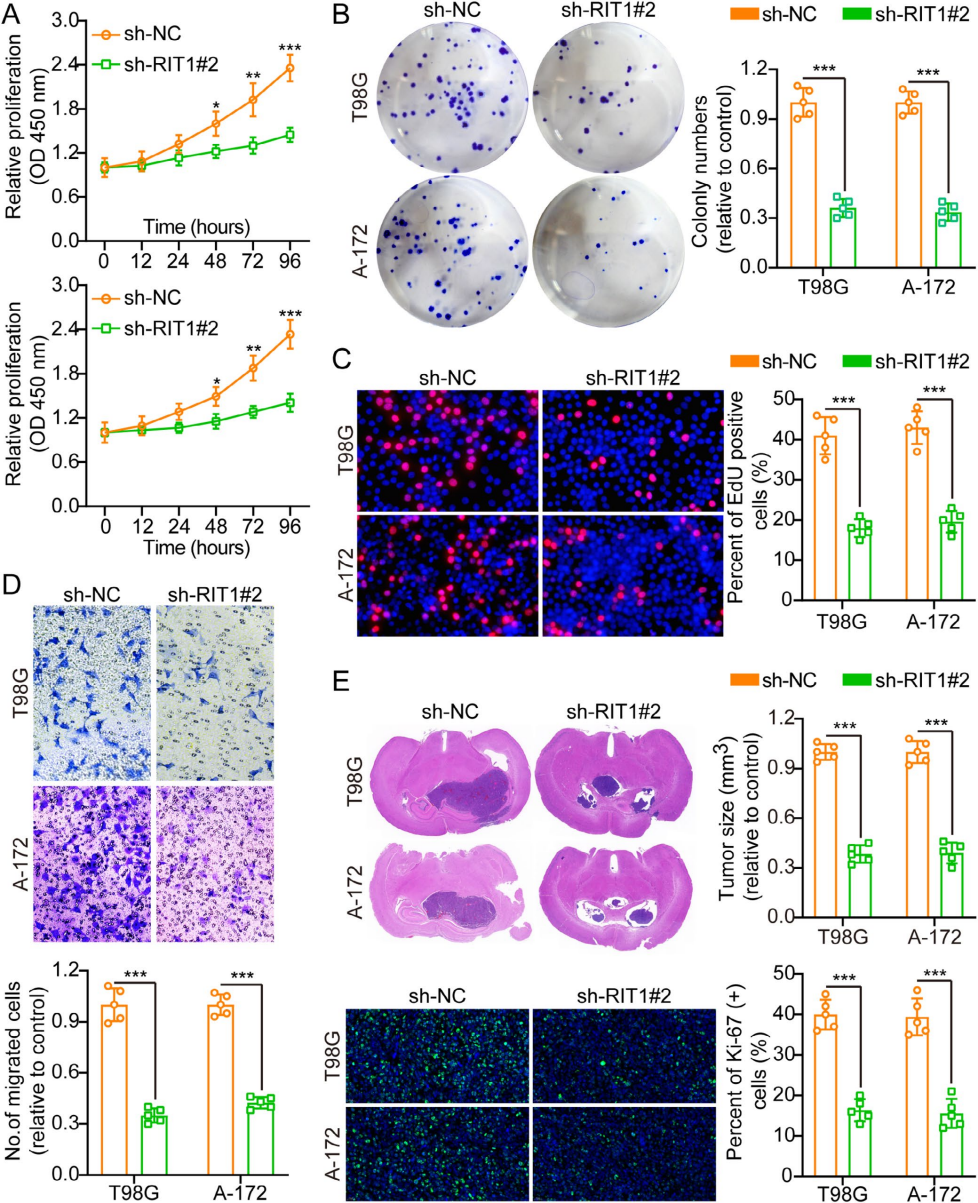
RIT1 knockdown inhibits glioma cell growth, migration, and invasion. (A) CCK‐8 measured cell growth curves between sh‐NC and sh‐RIT1#2, *n* = 5. (B) RIT1 knockdown inhibited colony formation and histogram analysis, *n* = 5. (C) EdU incorporation assays also indicated that RIT1 knockdown decreased EdU‐positive cells, inferring decreased proliferation of cells, *n* = 5. (D) Transwell migration assays indicate that RIT1 knockdown inhibited cell migration, *n* = 5. Scale bars: 50 μm. (E) Representative pictures and histogram of tumour size and Ki‐67 staining between sh‐NC and sh‐RIT1#2, *n* = 5. Data are presented as mean ± SD from five independent experiments. *p < 0.05, **p < 0.01 and ***p < 0.001.

The knockdown efficacy of RIT1 in T98G and A‐172 cells was validated using WB analysis (Figure S1E). Of the sh‐RNAs evaluated, sh‐RIT1#2 had the greatest knockdown efficacy and will consequently be employed in future research. The functional experiments, including the CCK8, colony formation, EdU and transwell assays, revealed that knocking down RIT1 significantly reduced the proliferation, migration and invasion of glioma cells (Figure 3B–D and Figure S1G), implying that RIT1 is crucial in promoting these activities. The flow cytometric analysis results demonstrated that RIT1 knockdown promotes apoptosis in glioma cells and inhibits cell cycle progression (Figure S2B). Furthermore, stable cell lines with RIT1 knockdown were implanted in situ into nude mice. Following a 3‐week duration, the mice were slain, and their brains were extracted, weighed and exposed to staining protocols. The IF for Ki‐67 was performed on the brain tumours, demonstrating that RIT1 knockdown led to reduced Ki‐67 expression relative to the control (Figure 3E). In addition, the results of TUNEL experiments also showed that overexpression of RIT1 inhibited cell apoptosis, while knockdown of RIT1 promoted cell apoptosis, which was consistent with the trend of flow cytometry results (Figure S3A). The results indicate that RIT1 suppression diminishes cellular proliferation in vivo.

### 3.4 RIT1 Promotes Glioma Growth by Activating PI3K‐AKT Signalling

**FIGURE 4:**
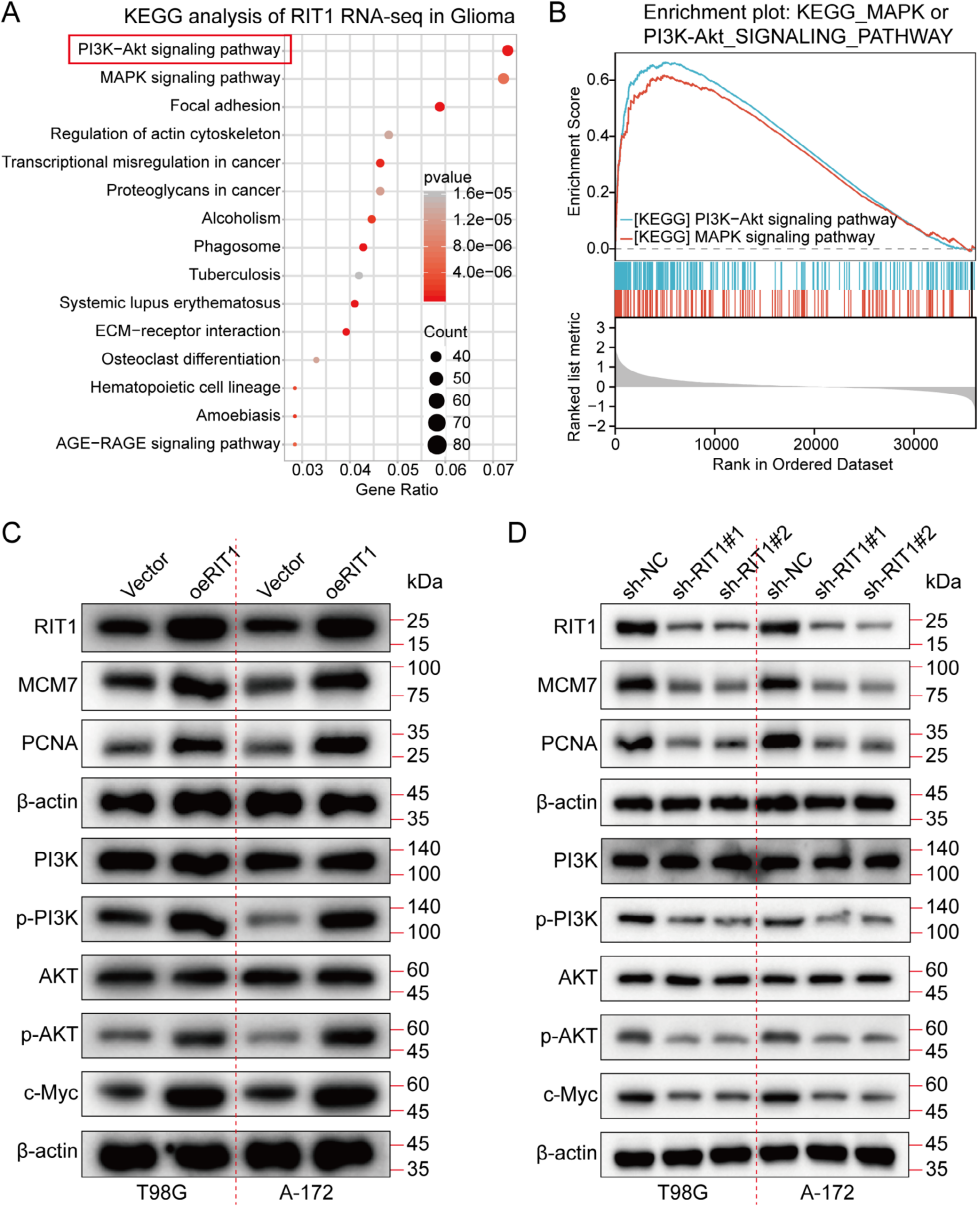
RIT1 activates the PI3K/AKT signalling pathway. (A, B) KEGG pathway analysis and GSEA results show that RIT1 is positively related to the PI3K/AKT signalling pathway. (C, D) The expression levels of RIT1, MCM7, PCNA, PI3K, p‐PI3K, AKT, p‐AKT and c‐Myc was analysed by WB in Vector, oeRIT1, sh‐NC, sh‐RIT1#1 and sh‐RIT1#2 groups.

A gene pathway enrichment analysis was performed to clarify the molecular processes by which RIT1 promotes glioma cell growth. The results demonstrated that RIT1 significantly influences PI3K‐AKT signalling (Figure 4A). Furthermore, Gene Set Enrichment Analysis (GSEA) demonstrated a significant correlation between RIT1 and PI3K‐AKT signalling pathways (Figure 4B). Furthermore, to validate the role of RIT1 in promoting tumour growth by activating PI3K‐AKT signalling, RIT1 knockdown and overexpression experiments were conducted in T98G and A‐172 cell lines, respectively. The WB was utilised to evaluate MCM7, PCNA, PI3K, phosphorylated PI3K (p‐PI3K), AKT, phosphorylated AKT (p‐AKT) and c‐Myc expression levels. The results demonstrated that RIT1 knockdown led to a downregulation of MCM7, PCNA, p‐PI3K, p‐AKT and c‐Myc, whereas RIT1 overexpression resulted in their upregulation (Figure 4C,D and Figure S4A). In addition, we analysed the TCGA database and found that RIT1 was positively correlated with molecules related to the PI3K‐AKT signalling pathway (Figure 4C,D; Figures S4B and S5A). Furthermore, we examined the three primary subtypes of the MAPK signalling pathway: ERK, JNK and p38 MAPK. The findings indicated that RIT1 exerted minimal influence on them (Figure S5B). The aforementioned data collectively demonstrate that RIT1 activates the PI3K/AKT/c‐Myc signalling pathway.

**FIGURE 5:**
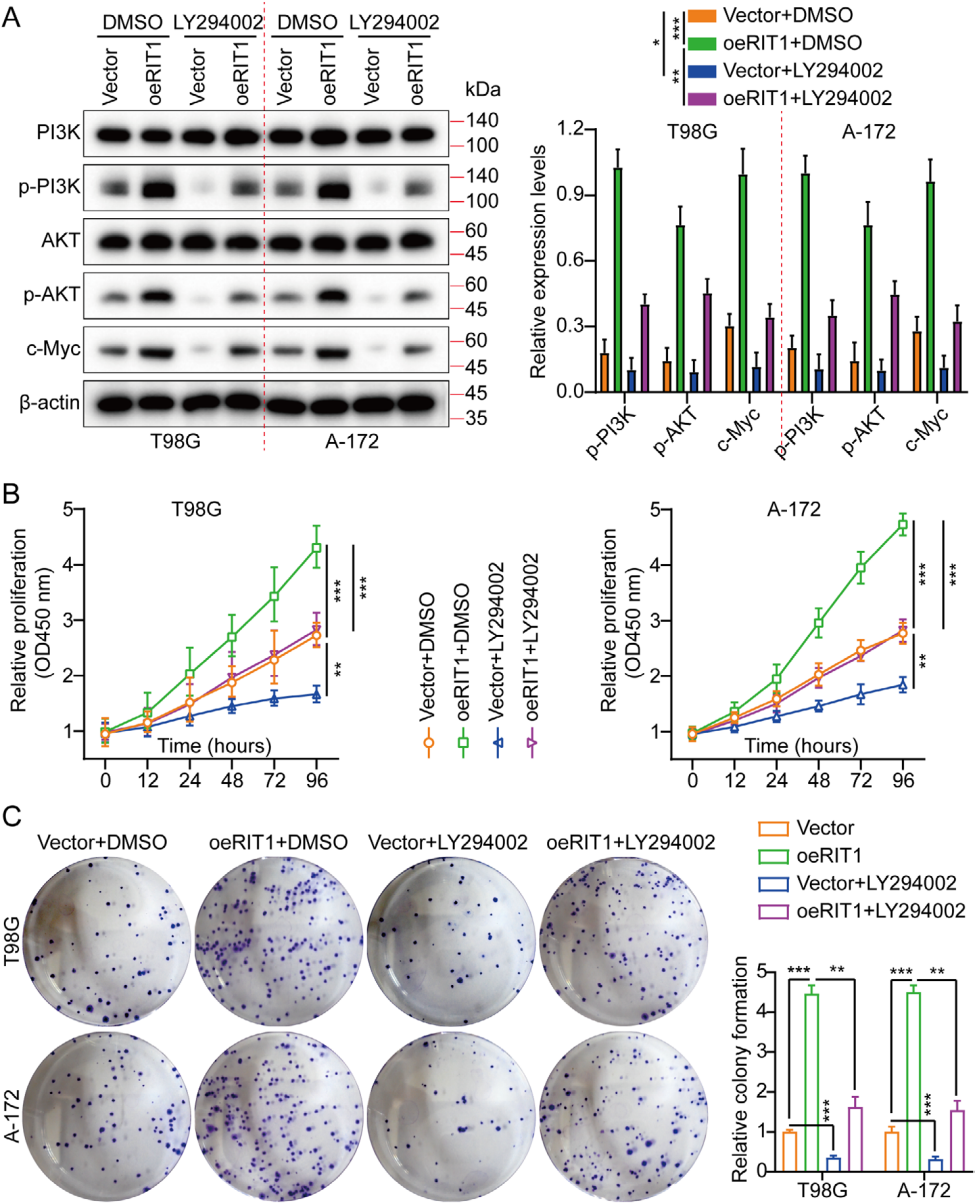
RIT1 promotes tumour growth by triggering the PI3K/AKT pathway. (A) PI3K, p‐PI3K, AKT, p‐AKT and c‐Myc levels were assessed by WB in Vector, oeRIT1, Vector + LY294002 and oeRIT1 + LY294002 groups. (B) CCK‐8 assay cell growth curves for various treatment groups. (C) Cell growth was assessed using a colony formation assay and histogram quantification across various treatment groups. Data are presented as mean ± SD from three independent experiments. *p < 0.05, **p < 0.01 and ***p < 0.001.

Besides, the study further explored the implication of RIT1 in enhancing cell proliferation through PI3K/AKT activation by treating T98G and A‐172 cells with the PI3K/AKT inhibitor LY294002 and examining the impacts of RIT1 overexpression on proliferation. Consistent with previous findings, RIT1 overexpression induced p‐PI3K, p‐AKT and c‐Myc without affecting PI3K and AKT levels (Figure 5A). However, this proliferative effect was attenuated upon treating T98G and A‐172 cells with the LY294002 (Figure 5B,C). Collectively, RIT1 may facilitate glioma progression by activating PI3K/AKT signalling.

### 3.5 RIT1 Promotes Glioma Growth via c‐Myc

The c‐Myc proteins are crucial for maintaining normal cellular function by regulating gene expression related to cell cycle progression, cell growth and apoptosis [27, 28]. Mutations or overexpression of c‐Myc genes have been associated with the disruption of cellular growth regulation besides being essential in cancer pathogenesis [29, 30]. The Myc gene is commonly overexpressed in multiple cancers [31]. In T98G and A‐172 cell lines, sh‐RNAs targeting c‐Myc were employed to further investigate its role in RIT1‐mediated glioma cell proliferation. Among the sh‐RNAs, sh‐c‐Myc#1 demonstrated the highest knockdown efficiency and will be utilised in subsequent studies (Figure S5C). Our prior data indicated that c‐Myc was downregulated by RIT1 knockdown, whereas it was enhanced by the overexpression of RIT1(Figure 4C,D and Figure S4A). Furthermore, to investigate whether RIT1 modulates c‐Myc expression by activating PI3K/AKT signalling, we overexpressed RIT1 and treated the cells with the PI3K/AKT inhibitor LY294002. The WB analysis results demonstrated that overexpression of RIT1 potentially elevates c‐Myc expression levels, whereas LY294002 attenuates the capacity of RIT1 overexpression to upregulate c‐Myc protein (Figure 5A).

**FIGURE 6:**
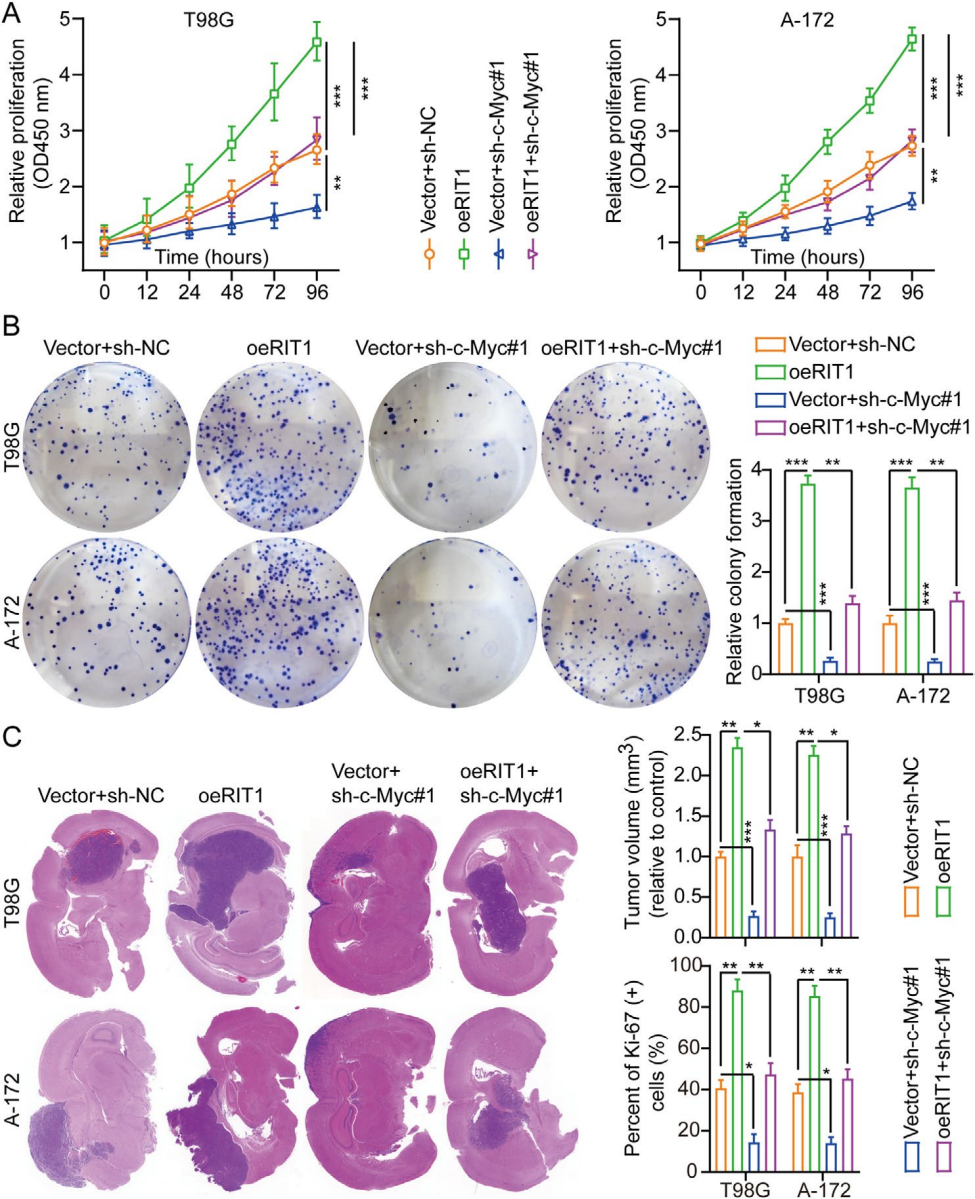
RIT1 promotes glioma growth via c‐Myc. (A) Cell growth curves were evaluated using the CCK‐8 assay across different treatment groups, *n* = 3. (B) Cell growth was evaluated using a colony formation assay and histogram analysis across different treatment groups, *n* = 3. (C) Representative frozen mouse brain tissue sections and tumour weight histograms across treatment groups, *n* = 3. The data were reported as the mean ± SD, derived from three autonomous experiments. *p < 0.05, **p < 0.01 and ***p < 0.001.

Furthermore, the silencing of c‐Myc mitigated the proliferative effects induced by RIT1 overexpression in T98G and A‐172 cells (Figure 6A,B and Figure S5D). These findings are consistent with in vitro studies, where c‐Myc silencing reduced the enhancement of cell growth caused by RIT1 overexpression in a glioma intracranial orthotopic transplantation tumour model in mice, more so than in the corresponding control groups (Figure 6C). The diminished expression of Ki‐67 in xenograft tumours within the c‐Myc silencing cohorts suggests that the silencing of c‐Myc may effectively mitigate the impact of RIT1 on cell proliferation (Figure S5E). In addition, TUNEL assay results showed that overexpression of RIT1 inhibited cell apoptosis, while knockdown of c‐Myc weakened the above effect (Figure S3B). Overall, these findings indicate that RIT1 facilitates tumour progression by activating PI3K/AKT/c‐Myc signalling.

### 3.6 RIT1 Is Directly Targeted by HIF‐1α

**FIGURE 7:**
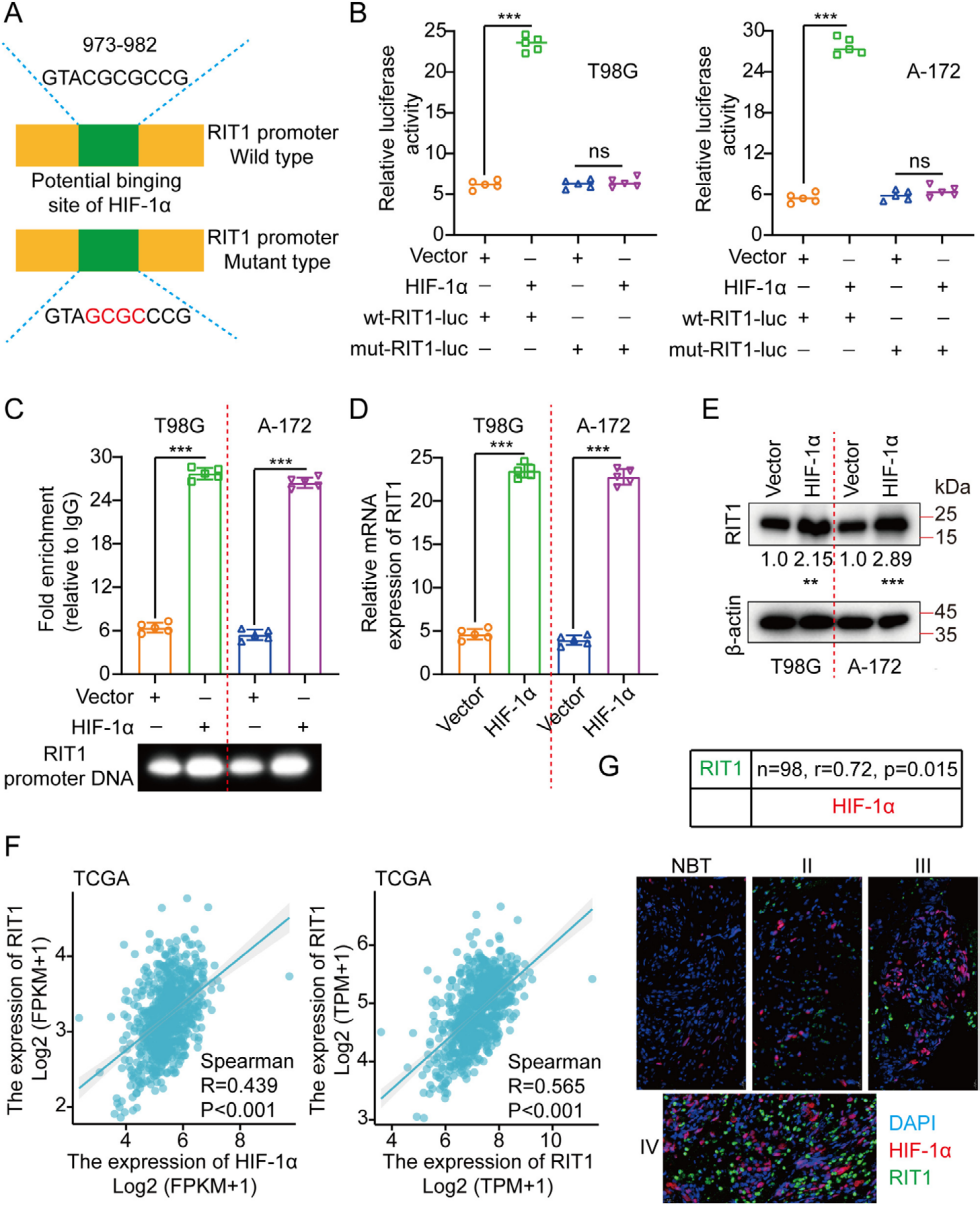
RIT1 is directly targeted by HIF‐1α. (A) Luciferase vectors, either wild‐type or mutant, were constructed based on HIF‐1α's potential binding site on the RIT1 promoter. (B) Luciferase activity was measured in T98G/A‐172 cells transfected with either wild‐type or mutant luciferase vectors and co‐transfected with expression plasmids, including empty vectors and HIF‐1α plasmids. (C) ChIP experiments were performed to study HIF‐1α's role in gene regulation, using IgG as an internal control for specificity. The DNA bound to HIF‐1α was PCR‐amplified with primers for the RIT1 promoter. (D, E) RIT1 expression was evaluated using qRT‐PCR and WB with ectopic HIF‐1α expression. (F) The results of TCGA database showed that HIF‐1α was positively correlated with RIT1. (G) Immunofluorescence staining of HIF‐1α and RIT1 in clinical samples and immune scoring were performed, and the results also showed a positive correlation between the two. The means ± SDs are provided (*n* = 5). **p < 0.01 and ***p < 0.001 according to two‐tailed Student *t*‐tests or one‐way ANOVA followed by Dunnett tests for several comparisons. Ns, no significant difference.

Hypoxia constitutes a significant environmental factor in glioma, frequently facilitating invasion, metastasis and malignancy [32, 33, 34]. Transcription factors exhibit a tendency to bind to specific DNA sequences to govern gene expression. Utilising the JASPAR database [35], it was observed that HIF‐1α binds to the RIT1 gene promoter. To elucidate the transcriptional regulation of RIT1 by HIF‐1α, luciferase vectors incorporating either wild‐type or mutated RIT1 promoters were constructed and transfected into T98G and A‐172 cells (Figure 7A). The luciferase assay demonstrated that the upregulation of HIF‐1α resulted in enhanced activity of the wild‐type (WT) RIT1 promoter, as evidenced by increased luciferase activity. In contrast, HIF‐1α overexpression did not affect the mutant (mut) RIT1 promoter activity (Figure 7B). Furthermore, Chromatin immunoprecipitation (ChIP) assays demonstrated HIF‐1α binding to the RIT1 promoter (Figure 7C). The HIF‐1α overexpression significantly elevated both RIT1 mRNA and protein levels (Figure 7D,E). Interestingly, we also found through analysis of the TCGA database that HIF‐1α and RIT1 are positively correlated in glioma tissues (Figure 7F). Finally, we used IF to stain HIF‐1α and RIT1 in clinical samples and performed immunoscores, and the results also showed that there was a positive correlation between the two (Figure 7G).

Ras‐like without CAAX 1 (RIT1), a small GTPase family member, is similar to Ras protein but lacks the CAAX sequence, so it has a unique role in signal transduction [8, 11]. The RIT1 has been shown to affect the function of CD8^+^ T cells in the tumour microenvironment [36], but its specific mechanism is still under exploration. Interestingly, we also found that RIT1 was inversely correlated with CD8^+^ T cells by analysing the glioma TCGA database (Figure S6A). Finally, we used IF to stain RIT1 and CD8^+^ T cells in clinical samples and performed immune scoring, and the results also showed a negative correlation between the two (Figure S6B).

## 4 Discussion

Glioma constitutes a primary tumour originating from glial cells in the brain and a prevalent intracranial tumour [2, 7, 37]. According to WHO classification, gliomas can be divided into grades I–IV, with grades I and II being low‐grade gliomas (LGG) and grades III and IV being high‐grade gliomas (HGG). In particular, HGGs such as GBM are highly invasive and fast‐growing, often invading surrounding healthy tissues and difficult to remove by surgery completely [38]. In addition, their cells are highly heterogeneous and prone to drug resistance, making treatment more difficult. The prognosis for patients with high‐grade gliomas is poor, especially for GBMs, where the survival period is often only 1–2 years, and the 5‐year survival rate is extremely low [38, 39]. It is precisely because of the high invasiveness, difficulty in curing, and serious impact on the function of the central nervous system that glioma has become one of the most challenging tumours in clinical practice, and it is urgent for us to study its occurrence and development mechanism further.

RIT1 is a Ras family GTPase belonging to the Ras subfamily of the small GTPase family. RIT1 gene mutations display a close connection with tumour occurrence and development [40], especially in some malignant tumours [9, 11], showing an important carcinogenic effect [41]. Mutations in RIT1 usually occur in the early stages of cancer and are driver mutations that can activate downstream signalling pathways to promote cell proliferation, migration, metastasis and anti‐apoptosis [16]. Studies have found that mutations in RIT1 are mainly concentrated in its GTP‐binding domain, which keeps it in an activated state, leading to excessive cell growth and malignant transformation [42]. RIT1 mutation has a high mutation frequency in some non‐small cell lung cancers [43] (especially lung adenocarcinoma). Its mutation can lead to excessive activation of signalling pathways such as EGFR, thereby promoting the growth of tumour cells [17]. Studies have shown that RIT1 mutations are also common in acute myeloid leukaemia, which can enhance leukaemia cell survival and proliferation, further exacerbating the disease [44]. The RIT1 mutations have been reported to promote cell proliferation and survival via Ras‐MAPK/ERK signalling whose activation is crucial in tumour occurrence and development [40]. RIT1 activation can also regulate cell survival and metabolism via PI3K/AKT signalling, making cancer cells more adaptable and anti‐apoptotic [15, 45]. There are also reports indicating that the role of RIT1 in cytoskeleton reorganisation can enhance cell migration and invasion capabilities and promote tumour metastasis. Our results align with those reported previously, showing that RIT1 promoted glioma growth, migration and invasion by activating PI3K/AKT signalling (Figures 2, 3, 4, 5). Because of the carcinogenic effect of RIT1 mutations in certain tumours, inhibiting its function may become a new treatment strategy. Drugs targeting RIT1 downstream signalling pathways (MAPK/ERK and PI3K/AKT) have shown effects in some tumours [46, 47]. Additionally, targeted therapy research for RIT1 is also gradually underway, hoping to develop inhibitors with strong specificity and low toxicity to inhibit the carcinogenic effect of RIT1.

The PI3K/AKT signalling pathway is a critical intracellular signal transduction pathway widely involved in cell proliferation, growth, survival, metabolism and migration [48]. When signalling molecules (such as growth factors) bind to cell membrane receptors (such as RTK or GPCR), PI3K (phosphatidylinositol 3‐kinase) is activated. The PI3K converts PIP2 (phosphatidylinositol 4,5‐bisphosphate) into PIP3 (phosphatidylinositol 3,4,5‐triphosphate), which is an important signalling molecule [49]. PIP3 acts as a second messenger, attracting PH domain‐containing AKT (termed protein kinase B, PKB) and PDK1 (3‐phosphoinositide‐dependent protein kinase 1) to the cell membrane, allowing AKT to be phosphorylated and fully activated. Activated AKT can regulate cell growth, metabolism and survival by phosphorylating a variety of downstream targets [50] (such as mTOR, GSK‐3β, FOXO transcription factors and c‐Myc proteins, among others), among which mTOR and c‐Myc is a key downstream effector molecule responsible for promoting protein synthesis, cell growth and metabolism. The c‐Myc is a vital transcription factor and a Myc gene family member (including c‐Myc, N‐Myc and L‐Myc). It mainly regulates cell growth, proliferation, metabolism and apoptosis. The c‐Myc initiates the transcription of downstream genes by binding to the E‐box sequence, involving many signalling pathways, such as cell cycle regulation, nucleic acid metabolism, and energy metabolism, among others [51]. High c‐Myc expression is closely correlated with various cancers (such as breast cancer [52], liver cancer [53] and lymphoma [54]) because it can enhance rapid cell proliferation and impede cell apoptosis. Due to its key role in tumours, c‐Myc has become one of the important targets for anti‐cancer treatment. Our findings align with previously reported data. Additionally, our experimental results demonstrated that RIT1 facilitates glioma aggressiveness by activating PI3K/AKT/c‐Myc signalling (Figures 2, 3, 4, 5).

Oxygen is the first element of human life, and the balance of oxygen concentration in the internal environment is a necessary condition for the body to carry out normal aerobic metabolism [55]. Hypoxia is a strong stress on the body and cells. Cells engage in several physiological and pathological processes by precisely modulating particular coding gene or non‐coding RNA expression via oxygen receptors and signal transduction pathways [56]. Hypoxia‐inducible Factors (HIFs) are part of the basic helix–loop–helix Per‐Arnt‐Sim (bHLH‐PAS) transcription factor superfamily. They function as heterodimers composed of the oxygen‐sensitive HIF‐α subunit and the constitutively expressed HIF‐β subunit (HIF‐1β). The HIFα subunit includes three subtypes: HIF‐1α, HIF‐2α and HIF‐3α. Currently, HIF‐1α is the most intensively studied and has the most prominent function [56]. The regulation of HIF activity is mainly achieved by regulating the protein stability of the HIF‐α subunit, and the α subunit protein stability is strictly governed by oxygen‐dependent hydroxylation. Under normoxic conditions, HIF‐1α cannot stably persist and is perpetually destroyed to sustain a low basal level. Prolyl Hydroxylase Domain (PHD) catalyses proline residues 402 and 564 hydroxylation in the HIF‐1α subunit, which is identified by the E3 ubiquitin ligase complex including the tumour suppressor protein von Hippel–Lindau (pVHL), causing its swift degradation via the ubiquitin‐proteasome pathway. In addition, under normoxic conditions, the factor Inhibiting HIF1 (FIH1) promotes asparagine hydroxylation at position 804 of HIF‐1α, thereby inhibiting its binding to P300 and inhibiting the transcriptional activation function of HIF‐1α. In hypoxic conditions, the activities of PHD and FIH1 hydroxylases are suppressed, leading to the nuclear HIF‐1α translocation, where it associates with HIF‐1β to create a transcription complex [57]. This complex then binds to the hypoxia‐responsive element (HRE) in the promoter region of target genes, thereby triggering the transcriptional expression of numerous downstream genes and engaging across multiple functions, both physiological and pathological. Besides being influenced by oxygen concentration, HIF‐1α is modulated by various factors, including the inhibitory role of the antisense transcription factor aHIF‐1α on HIF‐1α gene transcription. Additionally, specific growth factors, inflammatory mediators, as well as oncogenes may impact the stability of the HIF‐1α protein via signalling pathways such as PI3K/AKT and ERK1/2 [58]. A range of microRNAs has been reported to regulate HIF1. A multitude of research has demonstrated that HIF‐1α possesses biological roles, including the promotion of angiogenesis, regulation of homeostasis, modulation of circadian rhythms, induction of autophagy and programmed cell death, as well as facilitation of mesenchymal stem cell self‐renewal and differentiation. The expression level is frequently elevated abnormally in several main or secondary malignant tumour tissues [59]. It has emerged as a biomarker and prospective target for diagnostic purposes, targeted therapy, as well as prognostic assessment of numerous diseases [60]. Our investigations have indicated that HIF‐1α enhances the transcriptional activity and expression level of RIT1 (Figure 7). In general, hypoxia can occur in many physiological and pathological processes, and HIF‐1α has a pivotal regulatory role in hypoxic injury as well as cell, tissue and organ adaptation. The HIF‐1α is a double‐edged sword in the complex physiological and pathological processes of the body. Research on related molecular mechanisms will help guide its application as a molecular marker or drug target in clinical diagnosis and treatment.

Our findings indicate that RIT1 promotes glioma cell proliferation by activating PI3K/AKT/c‐Myc signalling (Figure S7). This emphasises the promise of RIT1 as a therapeutic target for glioma and shows the necessity for more research to clarify its particular molecular mechanisms and assess its therapeutic relevance.

## Author Contributions

**Zhen Liu:** conceptualization (equal), data curation (equal), formal analysis (equal), investigation (lead), methodology (equal), project administration (equal), software (equal), supervision (equal), validation (equal), visualization (equal), writing – original draft (equal), writing – review and editing (equal). **Hao‐dong Jiang:** conceptualization (equal), data curation (equal), formal analysis (equal), investigation (equal), methodology (equal), project administration (equal), resources (equal), software (equal), supervision (equal), validation (supporting), visualization (equal), writing – original draft (equal), writing – review and editing (equal). **Hao‐yuan Kan:** conceptualization (supporting), data curation (equal), formal analysis (supporting), investigation (equal), methodology (equal), project administration (equal), resources (supporting), software (equal), supervision (equal), validation (equal), visualization (equal), writing – original draft (equal), writing – review and editing (equal). **Li Zhang:** conceptualization (supporting), data curation (equal), formal analysis (supporting), methodology (supporting), resources (supporting), software (supporting), visualization (equal). **Yu‐xin Rao:** conceptualization (equal), data curation (supporting), formal analysis (supporting), funding acquisition (supporting), methodology (equal), software (supporting), visualization (equal). **Xiao‐bing Jiang:** conceptualization (equal), data curation (equal), formal analysis (equal), funding acquisition (equal), investigation (equal), methodology (equal), project administration (equal), resources (equal), software (equal), supervision (supporting), validation (supporting), visualization (supporting), writing – original draft (equal), writing – review and editing (equal). **Ming‐hui Li:** conceptualization (equal), data curation (equal), formal analysis (equal), funding acquisition (equal), investigation (equal), methodology (equal), project administration (lead), resources (equal), software (equal), supervision (equal), validation (equal), visualization (equal), writing – original draft (equal), writing – review and editing (equal). **Qi Wang:** conceptualization (equal), data curation (equal), formal analysis (equal), funding acquisition (equal), investigation (equal), methodology (equal), project administration (equal), resources (equal), supervision (equal), validation (equal), visualization (equal), writing – original draft (lead), writing – review and editing (lead).

## Ethics Statement

This study was reviewed and approved by the Ethical Board at Tongji Medical College of Huazhong University of Science and Technology.

## Conflicts of Interest

The authors declare no conflicts of interest.

## Supporting information


Data S1.

## Data Availability

The datasets utilised in this investigation can be obtained from the corresponding author upon an adequate inquiry.

## References

[1] D. Schiff , “Headway Against Brain Tumors With Molecular Targeting of IDH‐Mutant Gliomas,” New England Journal of Medicine 389, no. 7 (2023): 653–654, 10.1056/NEJMe2305639. 37585632

[2] F. Klemm , R. R. Maas , R. L. Bowman , et al., “Interrogation of the Microenvironmental Landscape in Brain Tumors Reveals Disease‐Specific Alterations of Immune Cells,” Cell 181, no. 7 (2020): 1643–1660, 10.1016/j.cell.2020.05.007. 32470396 PMC8558904

[3] A. Narayanan and S. Turcan , “The Magnifying GLASS: Longitudinal Analysis of Adult Diffuse Gliomas,” Cell 180, no. 3 (2020): 407–409, 10.1016/j.cell.2020.01.016. 32032515

[4] M. E. Hegi , A. C. Diserens , T. Gorlia , et al., “MGMT Gene Silencing and Benefit From Temozolomide in Glioblastoma,” New England Journal of Medicine 352, no. 10 (2005): 997–1003, 10.1056/NEJMoa043331. 15758010

[5] J. E. Eckel‐Passow , D. H. Lachance , A. M. Molinaro , et al., “Glioma Groups Based on 1p/19q, IDH, and TERT Promoter Mutations in Tumors,” New England Journal of Medicine 372, no. 26 (2015): 2499–2508, 10.1056/NEJMoa1407279. 26061753 PMC4489704

[6] M. Touat , Y. Y. Li , A. N. Boynton , et al., “Mechanisms and Therapeutic Implications of Hypermutation in Gliomas,” Nature 580, no. 7804 (2020): 517–523, 10.1038/s41586-020-2209-9. 32322066 PMC8235024

[7] R. Drexler , R. Khatri , T. Sauvigny , et al., “A Prognostic Neural Epigenetic Signature in High‐Grade Glioma,” Nature Medicine 30, no. 6 (2024): 1622–1635, 10.1038/s41591-024-02969-w. PMC11186787 38760585

[8] P. Castel , A. Cheng , A. Cuevas‐Navarro , et al., “RIT1 Oncoproteins Escape LZTR1‐Mediated Proteolysis,” Science 363, no. 6432 (2019): 1226–1230, 10.1126/science.aav1444. 30872527 PMC6986682

[9] A. Vichas , A. K. Riley , N. T. Nkinsi , et al., “Integrative Oncogene‐Dependency Mapping Identifies RIT1 Vulnerabilities and Synergies in Lung Cancer,” Nature Communications 12, no. 1 (2021): 4789, 10.1038/s41467-021-24841-y. PMC8352964 34373451

[10] A. H. Berger , M. Imielinski , F. Duke , et al., “Correction to: Oncogenic RIT1 Mutations in Lung Adenocarcinoma,” Oncogene 41, no. 19 (2022): 2788, 10.1038/s41388-022-02300-2. 35418694 PMC9828274

[11] Y. Su , H. Lin , J. Yu , et al., “RIT1 Regulates Mitosis and Promotes Proliferation by Interacting With SMC3 and PDS5 in Hepatocellular Carcinoma,” Journal of Experimental & Clinical Cancer Research 42, no. 1 (2023): 326, 10.1186/s13046-023-02892-x. 38017479 PMC10685607

[12] F. Xu , S. Sun , S. Yan , H. Guo , M. Dai , and Y. Teng , “Elevated Expression of RIT1 Correlates With Poor Prognosis in Endometrial Cancer,” International Journal of Clinical and Experimental Pathology 8, no. 9 (2015): 10315–10324. 26617739 PMC4637554

[13] A. Sadik , L. F. Somarribas Patterson , S. Ozturk , et al., “IL4I1 Is a Metabolic Immune Checkpoint That Activates the AHR and Promotes Tumor Progression,” Cell 182, no. 5 (2020): 1252–1270 e34, 10.1016/j.cell.2020.07.038. 32818467

[14] S. Chen , R. S. Vedula , A. Cuevas‐Navarro , et al., “Impaired Proteolysis of Noncanonical RAS Proteins Drives Clonal Hematopoietic Transformation,” Cancer Discovery 12, no. 10 (2022): 2434–2453, 10.1158/2159-8290.CD-21-1631. 35904492 PMC9533010

[15] Y. F. Feng , Y. Y. Lei , J. B. Lu , et al., “RIT1 Suppresses Esophageal Squamous Cell Carcinoma Growth and Metastasis and Predicts Good Prognosis,” Cell Death & Disease 9, no. 11 (2018): 1085, 10.1038/s41419-018-0979-x. 30348939 PMC6197279

[16] A. Cuevas‐Navarro , M. Wagner , R. Van , et al., “RAS‐Dependent RAF‐MAPK Hyperactivation by Pathogenic RIT1 Is a Therapeutic Target in Noonan Syndrome‐Associated Cardiac Hypertrophy,” Science Advances 9, no. 28 (2023): eadf4766, 10.1126/sciadv.adf4766. 37450595 PMC10348673

[17] Cancer Genome Atlas Research N , “Comprehensive Molecular Profiling of Lung Adenocarcinoma,” Nature 511, no. 7511 (2014): 543–550, 10.1038/nature13385. 25079552 PMC4231481

[18] P. Arlotta , B. J. Molyneaux , J. Chen , J. Inoue , R. Kominami , and J. D. Macklis , “Neuronal Subtype‐Specific Genes That Control Corticospinal Motor Neuron Development In Vivo,” Neuron 45, no. 2 (2005): 207–221, 10.1016/j.neuron.2004.12.036. 15664173

[19] E. Mohamed , A. Kumar , Y. Zhang , et al., “PI3K/AKT/mTOR Signaling Pathway Activity in IDH‐Mutant Diffuse Glioma and Clinical Implications,” Neuro‐Oncology 24, no. 9 (2022): 1471–1481, 10.1093/neuonc/noac064. 35287169 PMC9435510

[20] A. S. Guerreiro , S. Fattet , B. Fischer , et al., “Targeting the PI3K p110alpha Isoform Inhibits Medulloblastoma Proliferation, Chemoresistance, and Migration,” Clinical Cancer Research 14, no. 21 (2008): 6761–6769, 10.1158/1078-0432.CCR-08-0385. 18980969

[21] J. Li , K. Wang , C. Yang , et al., “Tumor‐Associated Macrophage‐Derived Exosomal LINC01232 Induces the Immune Escape in Glioma by Decreasing Surface MHC‐I Expression,” Advance Science (Weinh) 10, no. 17 (2023): e2207067, 10.1002/advs.202207067. PMC10265094 37097629

[22] Y. Zeng , C. Xiong , N. Tang , et al., “Erratum: FAM72A Promotes Glioma Progression by Regulating Mitophagy Through the Pink1/Parkin Signaling Pathway: Erratum,” Journal of Cancer 15, no. 6 (2024): 1733, 10.7150/jca.93189. 38370373 PMC10869974

[23] C. Jiang , Y. Zhou , L. Yan , et al., “A Prognostic NAD+ Metabolism‐Related Gene Signature for Predicting Response to Immune Checkpoint Inhibitor in Glioma,” Frontiers in Oncology 13 (2023): 1051641, 10.3389/fonc.2023.1051641. 36845744 PMC9945104

[24] J. He , K. Xue , F. Fan , et al., “KIAA0040 Enhances Glioma Growth by Controlling the JAK2/STAT3 Signalling Pathway,” Journal of Cellular and Molecular Medicine 28, no. 8 (2024): e18332, 10.1111/jcmm.18332. 38661644 PMC11044867

[25] J. Li , H. Yuan , H. Xu , H. Zhao , and N. Xiong , “Hypoxic Cancer‐Secreted Exosomal miR‐182‐5p Promotes Glioblastoma Angiogenesis by Targeting Kruppel‐Like Factor 2 and 4,” Molecular Cancer Research 18, no. 8 (2020): 1218–1231, 10.1158/1541-7786.MCR-19-0725. 32366676

[26] J. Li , T. Liao , H. Liu , et al., “Hypoxic Glioma Stem Cell‐Derived Exosomes Containing Linc01060 Promote Progression of Glioma by Regulating the MZF1/c‐Myc/HIF1alpha Axis,” Cancer Research 81, no. 1 (2021): 114–128, 10.1158/0008-5472.CAN-20-2270. 33158815

[27] A. Baluapuri , E. Wolf , and M. Eilers , “Target Gene‐Independent Functions of MYC Oncoproteins,” Nature Reviews. Molecular Cell Biology 21, no. 5 (2020): 255–267, 10.1038/s41580-020-0215-2. 32071436 PMC7611238

[28] E. Zlotorynski , “MYC in TOP Shape,” Nature Reviews. Molecular Cell Biology 23, no. 2 (2022): 92, 10.1038/s41580-021-00444-9. 34912109

[29] R. Dhanasekaran , A. Deutzmann , W. D. Mahauad‐Fernandez , A. S. Hansen , A. M. Gouw , and D. W. Felsher , “The MYC Oncogene—The Grand Orchestrator of Cancer Growth and Immune Evasion,” Nature Reviews. Clinical Oncology 19, no. 1 (2022): 23–36, 10.1038/s41571-021-00549-2. PMC9083341 34508258

[30] H. Fatma , S. K. Maurya , and H. R. Siddique , “Epigenetic Modifications of c‐MYC: Role in Cancer Cell Reprogramming, Progression and Chemoresistance,” Seminars in Cancer Biology 83 (2022): 166–176, 10.1016/j.semcancer.2020.11.008. 33220458

[31] M. J. Duffy , S. O'Grady , M. Tang , and J. Crown , “MYC as a Target for Cancer Treatment,” Cancer Treatment Reviews 94 (2021): 102154, 10.1016/j.ctrv.2021.102154. 33524794

[32] A. Sattiraju , S. Kang , B. Giotti , et al., “Hypoxic Niches Attract and Sequester Tumor‐Associated Macrophages and Cytotoxic T Cells and Reprogram Them for Immunosuppression,” Immunity 56, no. 8 (2023): 1825–1843, 10.1016/j.immuni.2023.06.017. 37451265 PMC10527169

[33] A. Rashidi , L. K. Billingham , A. Zolp , et al., “Myeloid Cell‐Derived Creatine in the Hypoxic Niche Promotes Glioblastoma Growth,” Cell Metabolism 36, no. 1 (2024): 62–77, 10.1016/j.cmet.2023.11.013. 38134929 PMC10842612

[34] J. H. Park , H. J. Kim , C. W. Kim , et al., “Tumor Hypoxia Represses Gammadelta T Cell‐Mediated Antitumor Immunity Against Brain Tumors,” Nature Immunology 22, no. 3 (2021): 336–346, 10.1038/s41590-020-00860-7. 33574616

[35] I. Rauluseviciute , R. Riudavets‐Puig , R. Blanc‐Mathieu , et al., “JASPAR 2024: 20th Anniversary of the Open‐Access Database of Transcription Factor Binding Profiles,” Nucleic Acids Research 52, no. D1 (2024): D174–D182, 10.1093/nar/gkad1059. 37962376 PMC10767809

[36] Y. Wakabayashi , H. Watanabe , J. Inoue , et al., “Bcl11b Is Required for Differentiation and Survival of Alphabeta T Lymphocytes,” Nature Immunology 4, no. 6 (2003): 533–539, 10.1038/ni927. 12717433

[37] K. R. Taylor and M. Monje , “Invasive Glioma Cells: The Malignant Pioneers That Follow the Current,” Cell 185, no. 16 (2022): 2846–2848, 10.1016/j.cell.2022.06.033. 35931016

[38] N. D. Mathewson , O. Ashenberg , I. Tirosh , et al., “Inhibitory CD161 Receptor Identified in Glioma‐Infiltrating T Cells by Single‐Cell Analysis,” Cell 184, no. 5 (2021): 1281–1298, 10.1016/j.cell.2021.01.022. 33592174 PMC7935772

[39] FDA Approves First IDH‐Targeted Glioma Drug,” Nature Biotechnology 42, no. 9 (2024): 1325, 10.1038/s41587-024-02408-8. 39271837

[40] L. Sun , S. Xi , Z. Zhou , et al., “Elevated Expression of RIT1 Hyperactivates RAS/MAPK Signal and Sensitizes Hepatocellular Carcinoma to Combined Treatment With Sorafenib and AKT Inhibitor,” Oncogene 41, no. 5 (2022): 732–744, 10.1038/s41388-021-02130-8. 34845378

[41] A. Lo , K. Holmes , S. Kamlapurkar , et al., “Multiomic Characterization of Oncogenic Signaling Mediated by Wild‐Type and Mutant RIT1,” Science Signaling 14, no. 711 (2021): eabc4520, 10.1126/scisignal.abc4520. 34846918 PMC8848860

[42] A. Cuevas‐Navarro , R. Van , A. Cheng , A. Urisman , P. Castel , and F. McCormick , “The RAS GTPase RIT1 Compromises Mitotic Fidelity Through Spindle Assembly Checkpoint Suppression,” Current Biology 31, no. 17 (2021): 3915–3924, 10.1016/j.cub.2021.06.030. 34237269 PMC8440430

[43] A. H. Berger , M. Imielinski , F. Duke , et al., “Oncogenic RIT1 Mutations in Lung Adenocarcinoma,” Oncogene 33, no. 35 (2014): 4418–4423, 10.1038/onc.2013.581. 24469055 PMC4150988

[44] I. Gomez‐Segui , H. Makishima , A. Jerez , et al., “Novel Recurrent Mutations in the RAS‐Like GTP‐Binding Gene RIT1 in Myeloid Malignancies,” Leukemia 27, no. 9 (2013): 1943–1946, 10.1038/leu.2013.179. 23765226 PMC8711127

[45] E. V. Rusyn , E. R. Reynolds , H. Shao , et al., “Rit, a Non‐Lipid‐Modified Ras‐Related Protein, Transforms NIH3T3 Cells Without Activating the ERK, JNK, p38 MAPK or PI3K/Akt Pathways,” Oncogene 19, no. 41 (2000): 4685–4694, 10.1038/sj.onc.1203836. 11032018

[46] E. Jasek‐Gajda , H. Jurkowska , M. Jasinska , and G. J. Lis , “Targeting the MAPK/ERK and PI3K/AKT Signaling Pathways Affects NRF2, Trx and GSH Antioxidant Systems in Leukemia Cells,” Antioxidants (Basel) 9, no. 7 (2020): 663, 10.3390/antiox9070633. 32709140 PMC7402140

[47] X. Peng , Y. Liu , S. Zhu , et al., “Co‐Targeting PI3K/Akt and MAPK/ERK Pathways Leads to an Enhanced Antitumor Effect on Human Hypopharyngeal Squamous Cell Carcinoma,” Journal of Cancer Research and Clinical Oncology 145, no. 12 (2019): 2921–2936, 10.1007/s00432-019-03047-2. 31620898 PMC11810336

[48] A. Glaviano , A. S. C. Foo , H. Y. Lam , et al., “PI3K/AKT/mTOR Signaling Transduction Pathway and Targeted Therapies in Cancer,” Molecular Cancer 22, no. 1 (2023): 138, 10.1186/s12943-023-01827-6. 37596643 PMC10436543

[49] N. Thapa , M. Chen , H. T. Horn , S. Choi , T. Wen , and R. A. Anderson , “Phosphatidylinositol‐3‐OH Kinase Signalling Is Spatially Organized at Endosomal Compartments by Microtubule‐Associated Protein 4,” Nature Cell Biology 22, no. 11 (2020): 1357–1370, 10.1038/s41556-020-00596-4. 33139939 PMC8647654

[50] P. Tiemin , M. Fanzheng , X. Peng , et al., “MUC13 Promotes Intrahepatic Cholangiocarcinoma Progression via EGFR/PI3K/AKT Pathways,” Journal of Hepatology 72, no. 4 (2020): 761–773, 10.1016/j.jhep.2019.11.021. 31837357

[51] A. Guo , H. Huang , Z. Zhu , et al., “cBAF Complex Components and MYC Cooperate Early in CD8^+^ T Cell Fate,” Nature 607, no. 7917 (2022): 135–141, 10.1038/s41586-022-04849-0. 35732731 PMC9623036

[52] D. Zimmerli , C. S. Brambillasca , F. Talens , et al., “MYC Promotes Immune‐Suppression in Triple‐Negative Breast Cancer via Inhibition of Interferon Signaling,” Nature Communications 13, no. 1 (2022): 6579, 10.1038/s41467-022-34000-6. PMC9630413 36323660

[53] L. D'Artista , A. A. Moschopoulou , I. Barozzi , et al., “MYC Determines Lineage Commitment in KRAS‐Driven Primary Liver Cancer Development,” Journal of Hepatology 79, no. 1 (2023): 141–149, 10.1016/j.jhep.2023.02.039. 36906109 PMC10330789

[54] A. Rosenwald , S. Bens , R. Advani , et al., “Prognostic Significance of MYC Rearrangement and Translocation Partner in Diffuse Large B‐Cell Lymphoma: A Study by the Lunenburg Lymphoma Biomarker Consortium,” Journal of Clinical Oncology 37, no. 35 (2019): 3359–3368, 10.1200/JCO.19.00743. 31498031

[55] H. Sies and D. P. Jones , “Reactive Oxygen Species (ROS) as Pleiotropic Physiological Signalling Agents,” Nature Reviews. Molecular Cell Biology 21, no. 7 (2020): 363–383, 10.1038/s41580-020-0230-3. 32231263

[56] P. Lee , N. S. Chandel , and M. C. Simon , “Cellular Adaptation to Hypoxia Through Hypoxia Inducible Factors and Beyond,” Nature Reviews. Molecular Cell Biology 21, no. 5 (2020): 268–283, 10.1038/s41580-020-0227-y. 32144406 PMC7222024

[57] L. M. Sanmarco , J. M. Rone , C. M. Polonio , et al., “Lactate Limits CNS Autoimmunity by Stabilizing HIF‐1alpha in Dendritic Cells,” Nature 620, no. 7975 (2023): 881–889, 10.1038/s41586-023-06409-6. 37558878 PMC10725186

[58] Z. H. Liao , H. Q. Zhu , Y. Y. Chen , et al., “The Epigallocatechin Gallate Derivative Y(6) Inhibits Human Hepatocellular Carcinoma by Inhibiting Angiogenesis in MAPK/ERK1/2 and PI3K/AKT/HIF‐1alpha/VEGF Dependent Pathways,” Journal of Ethnopharmacology 259 (2020): 112852, 10.1016/j.jep.2020.112852. 32278759

[59] J. Ni , X. Wang , A. Stojanovic , et al., “Single‐Cell RNA Sequencing of Tumor‐Infiltrating NK Cells Reveals That Inhibition of Transcription Factor HIF‐1alpha Unleashes NK Cell Activity,” Immunity 52, no. 6 (2020): 1075–1087, 10.1016/j.immuni.2020.05.001. 32445619

[60] Q. Li , Y. Ni , L. Zhang , et al., “HIF‐1alpha‐Induced Expression of m6A Reader YTHDF1 Drives Hypoxia‐Induced Autophagy and Malignancy of Hepatocellular Carcinoma by Promoting ATG2A and ATG14 Translation,” Signal Transduction and Targeted Therapy 6, no. 1 (2021): 76, 10.1038/s41392-020-00453-8. 33619246 PMC7900110

