# Supplementary material for: RIT1 Promotes the Proliferation of Gliomas Through the Regulation of the PI3K/AKT/c‐Myc Signalling Pathway
Source: J Cell Mol Med. 2025 Jan 20;29(2):e70362. doi: 10.1111/jcmm.70362 (PMC11745823; doi:10.1111/jcmm.70362)
Supplement: Supplementary file 1 — Data S1. [file JCMM-29-e70362-s001.docx]

**RIT1 promotes the proliferation of gliomas through the regulation of the PI3K/AKT/c-Myc signaling pathway**

Zhen Liu^1*^, Hao-dong Jiang^1*^, Hao-yuan Kan^1*^, Li Zhang^1^, Yu-xin Rao^1^, Xiao-bing Jiang^1#^,Ming-hui Li^2#^, Qi Wang^3#^

**Materials and Methods**

**Real-time quantitative RT-PCR (qRT-PCR)**

Total RNA was extracted from cells and tissues using TRIzol and TRIzol LS reagents (Life Technologies). miRNAs were reverse transcribed with the Mir-X™ miRNA First-Strand Synthesis Kit (Clontech, Mountain View, CA, USA). Real-time PCR was conducted using SYBR Green PCR Master Mix (Takara, Shiga, Japan) and primers from Table S5. mRNA levels were quantified using the 7500 Fast Real-Time PCR System (Applied Biosystems, Foster City, CA, USA), with GAPDH serving as the internal control.

Table S1**:** Correlation of the expression levels of RIT1 in glioma tissues with clinicopathologic features.

| Features | No. | RIT1 | | P-value |
| --- | --- | --- | --- | --- |
|  |  | Low | High |  |
| **Age(years)** |  | | | |
| <50 | 52 (53.1%) | 25 (25.5%) | 27 (27.6%) | 0.498 |
| >=50 | 46 (46.9%) | 19 (19.4%) | 27 (27.5%) |  |
| **Gender** |  | | | |
| Male | 48 (49.0%) | 21 (21.4%) | 27 (27.6%) | 0.807 |
| Female | 50 (51.0%) | 23 (23.5%) | 27 (27.5%) |  |
| **Tumor size, cm** |  | | | |
| <2 | 60 (61.2%) | 36 (36.7%) | 24 (24.5%) | P<0.001*** |
| >=2 | 38 (38.8%) | 8 (8.2%) | 30 (30.6%) |  |
| **Tumor location** |  | | | |
| Supratentorial | 40 (40.8%) | 19 (19.4%) | 21 (21.4%) | 0.679 |
| Subtentorial | 25 (59.2%) | 17 (25.5%) | 33 (33.7%) |  |
| **Karnofsky performance scale** |  | | | |
| <90 | 56 (57.1%) | 19 (19.4%) | 37 (37.7%) | p=0.012* |
| >=90 | 42 (42.9%) | 25 (25.5%) | 17 (17.4%) |  |
| **WHO grade** |  | | | |
| Low-grade(Ⅰ+Ⅱ) | 44 (44.9%) | 30 (30.6%) | 14 (14.3%) | P<0.001*** |
| High-grade(Ⅲ+Ⅳ) | 54 (55.1%) | 14 (14.3%) | 40 (40.8%) |  |
| **Tumor recurrence** |  | | | |
| No | 33 (33.7%) | 20 (20.4%) | 13 (13.3%) | p=0.025* |
| Yes | 65 (66.3%) | 24 (24.5%) | 41 (41.8%) |  |

statistics based on available data. ***<0.001. RIT1 high expression: score 8-16; low expression: score 0-7.

Table S2**:** Univariate and multivariate for clinicopathological features associated with various prognostic parameters of 98 glioma patients by Cox-regression analysis.

| Variables | Univariate analysis | | Multivariate analysis | |
| --- | --- | --- | --- | --- |
|  | HR (95%CI) | P-value | HR (95%CI) | P-value |
| WHO grade (I+II vs III+IV) | 2.538 (1.364-4.195) | P<0.001*** | 1.972 (1.132-2.964) | 0.005** |
| Tumor size (≥2cm vs ＜2cm) | 2.173  (1.286-3.957) | P<0.01** | 1.683 (1.176-2.586) | 0.016* |
| Tumor recurrence (Yes vs No) | 1.539 (1.328-3.695) | p=0.037* | 1.257 (0.972-3.217) | 0.086 |
| RIT1 (Low vs High) | 1.668  (1.053-2.834) | 0.021* | 1.187 (0.895-2.626) | 0.092 |

Table S3**:** Related antibodies involved

| Name | Description |
| --- | --- |
| Anti-RIT1 antibody | ab53720 (Abcam, Cambridge, USA) |
| Anti-β-Actin antibody | #8457 (Cell Signaling Technology, Beverly, MA, USA) |
| Anti-MCM7 antibody | ab52489 (Abcam, Cambridge, USA) |
| Anti-PCNA antibody | ab29 (Abcam, Cambridge, USA) |
| Anti-PI3K antibody | ab302958 (Abcam, Cambridge, USA) |
| Anti-AKT antibody | ab8805 (Abcam, Cambridge, USA) |
| Anti-c-Myc antibody | 10828-1-AP (Proteintech, Wuhan, China ) |
| Anti-HIF-1α antibody | 20960-1-AP (Proteintech, Wuhan, China ) |
| Anti-Ki-67 antibody | #9129 (Cell Signaling Technology, Beverly, MA, USA) |

Table S4**:** The specific sh-RNAs sequences utilized in this study

| **Gene** | **Primer** | **Sequence(5′-3′)** |
| --- | --- | --- |
| sh-RIT1#1 | forward | CGTCGAAGTTTCCATGAAGTT |
| sh-RIT1#2 | forward | CAGTGTATGGAAGAGGCTAAA |
| sh-c-Myc#1 | forward | TAATGATAACCAGAACTTGCT |
| sh-c-Myc#2 | forward | CACCACCAGCACACGGAACTA |

Table S5**:** The Primer sequences in this study

| **Gene** | **Primer** | **Sequence(5′-3′)** |
| --- | --- | --- |
| RIT1 | forward | TTCATCAGCCACCGATTCCC |
|  | reverse | GCAGGCTCATCATCAATACGGA |
| c-Myc | forward | GGCTCCTGGCAAAAGGTCA |
|  | reverse | CTGCGTAGTTGTGCTGATGT |
| GAPDH | forward | GAGTCAACGGATTTGGTCGT |
|  | reverse | TTGATTTTGGAGGGATCTCG |

**Figure Legends**

**
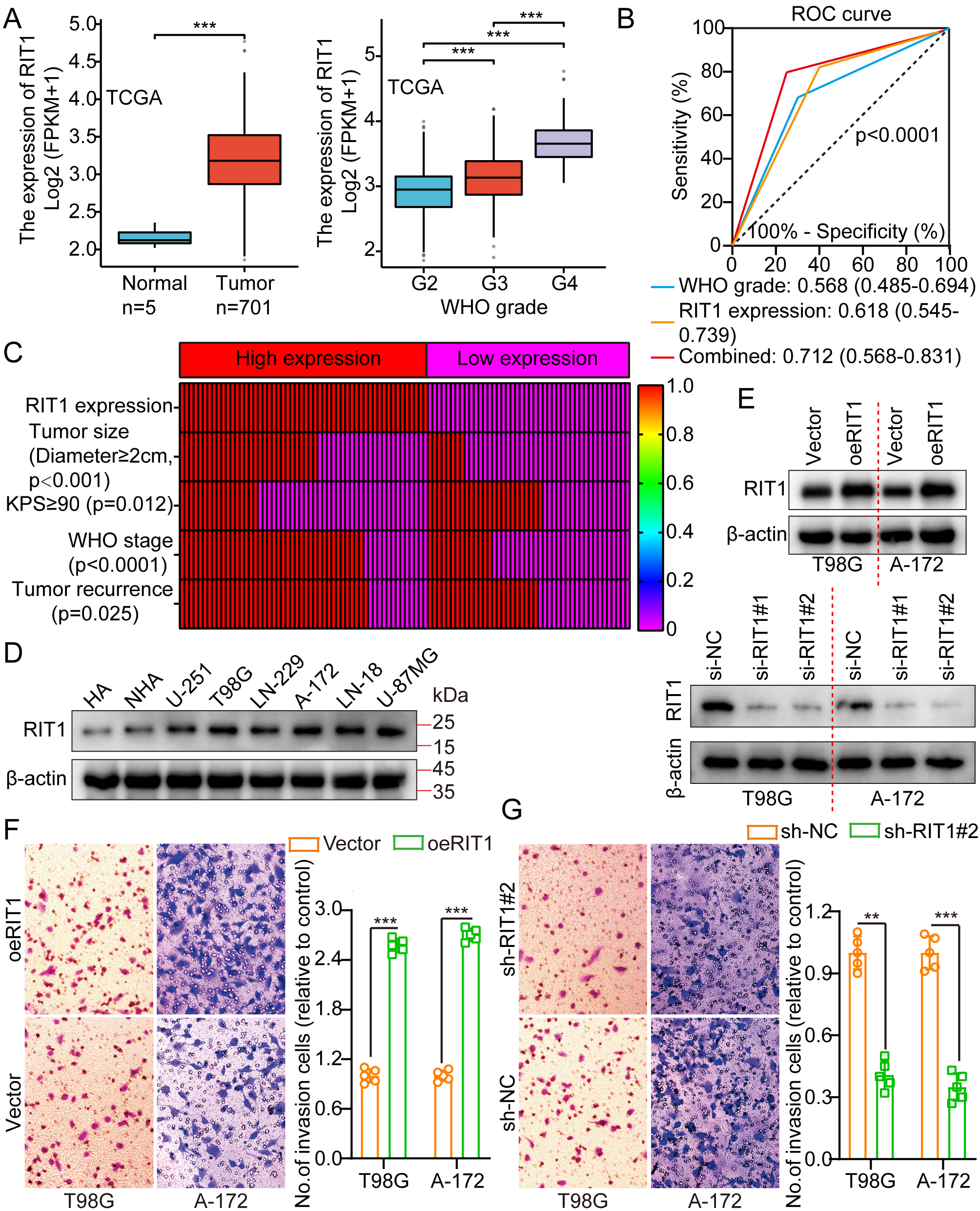
**

**Supplementary Figure 1: Bioinformatics analysis of RIT1.**

**A.** Analysis of the TCGA database reveals that RIT1 expression is elevated in tumor tissues and rises with tumor grade. **B.** ROC analysis of RIT1, WHO, and combined models for predicting clinical outcomes. **C.** The heatmap shows the link between various clinical traits and tumors with high or low RIT1 expression. **D.** WB to compare RIT1 expression levels in HA, NHA, U-251, T98G, LN-229, A-172, LN-18 and U-87MG cells. **E.** Validation of RIT1 overexpression and knockdown efficiency via WB. **F.** Transwell invasion assays indicate that RIT1 overexpression enhances cell invasion, n=5. Scale bars: 50 µm. **G.** Transwell invasion assays indicate that RIT1 knockdown inhibited cell invasion, n=5. Scale bars: 50 µm. Statistical significance was tested using one-way ANOVA (Dunnett’s tests) for several comparisons and two-tailed t-tests. **P* < 0.05, ***P* < 0.01 and ****P* < 0.001.


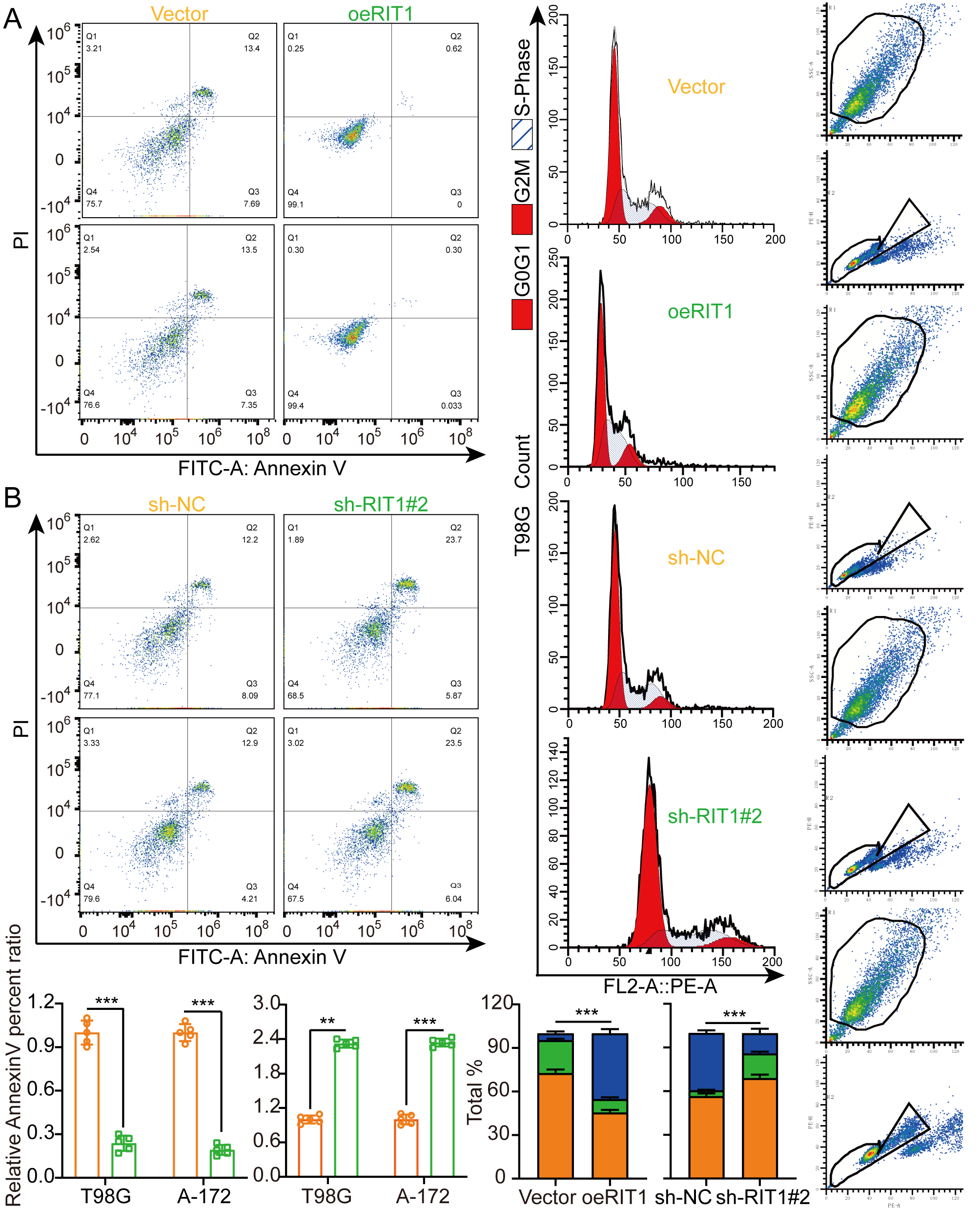


**Supplementary Figure 2: RIT1 inhibits apoptosis and promotes cell cycle progression.**

**A.** Flow cytometry data showed that overexpression of RIT1 inhibited apoptosis and promoted cell cycle progression, n=5. **B.** Flow cytometry data showed that knockdown of RIT1 promoted apoptosis and inhibited cell cycle progression, n=5. Statistical significance was tested using one-way ANOVA (Dunnett’s tests) for several comparisons and two-tailed t-tests. ***P* < 0.01 and ****P* < 0.001.


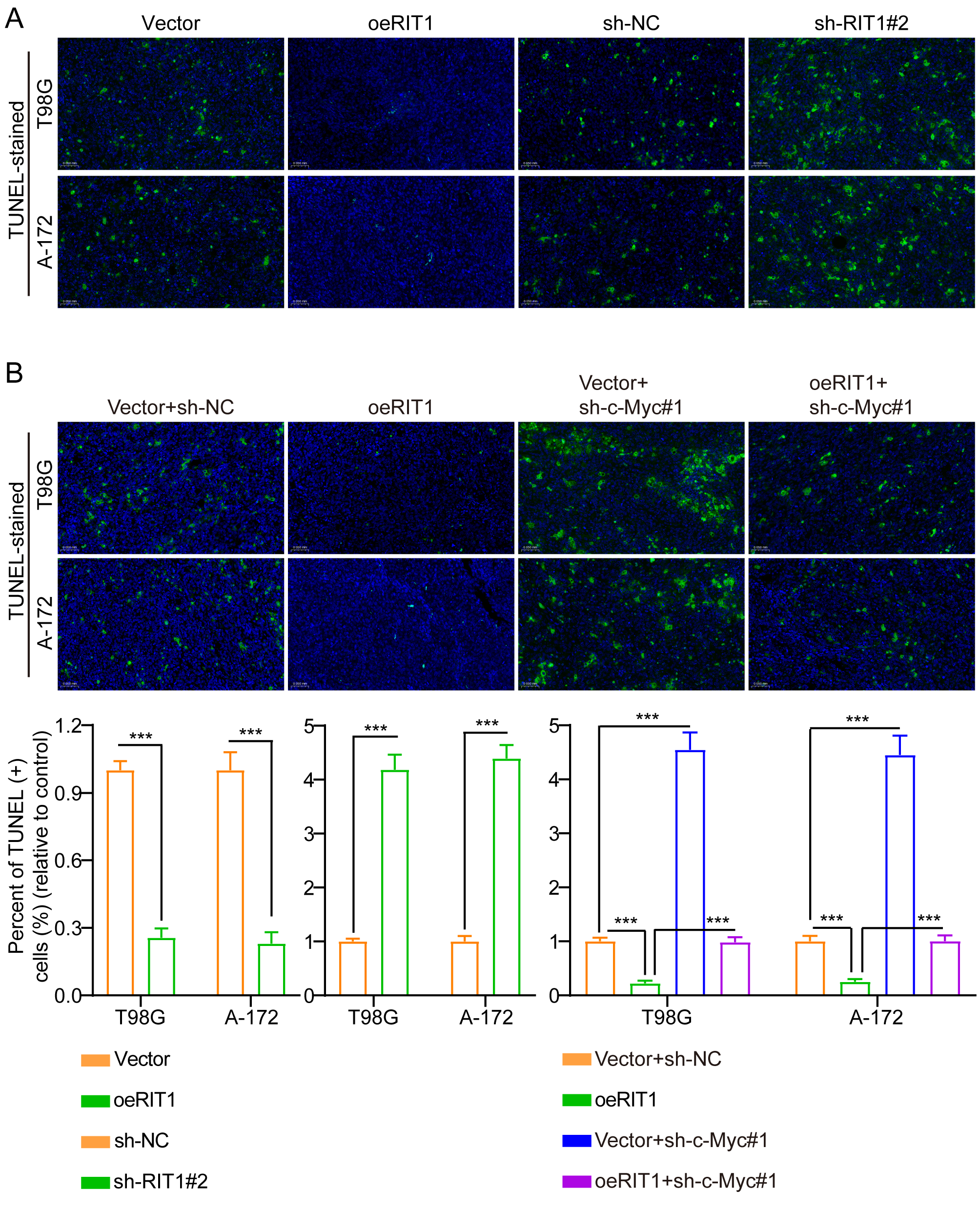


**Supplementary Figure 3: RIT1 inhibits apoptosis.**

**A-B.** Representative pictures of TUNEL-stained at different treatment groups, n=3. Statistical significance was tested using one-way ANOVA (Dunnett’s tests) for several comparisons and two-tailed t-tests. ****P* < 0.001.


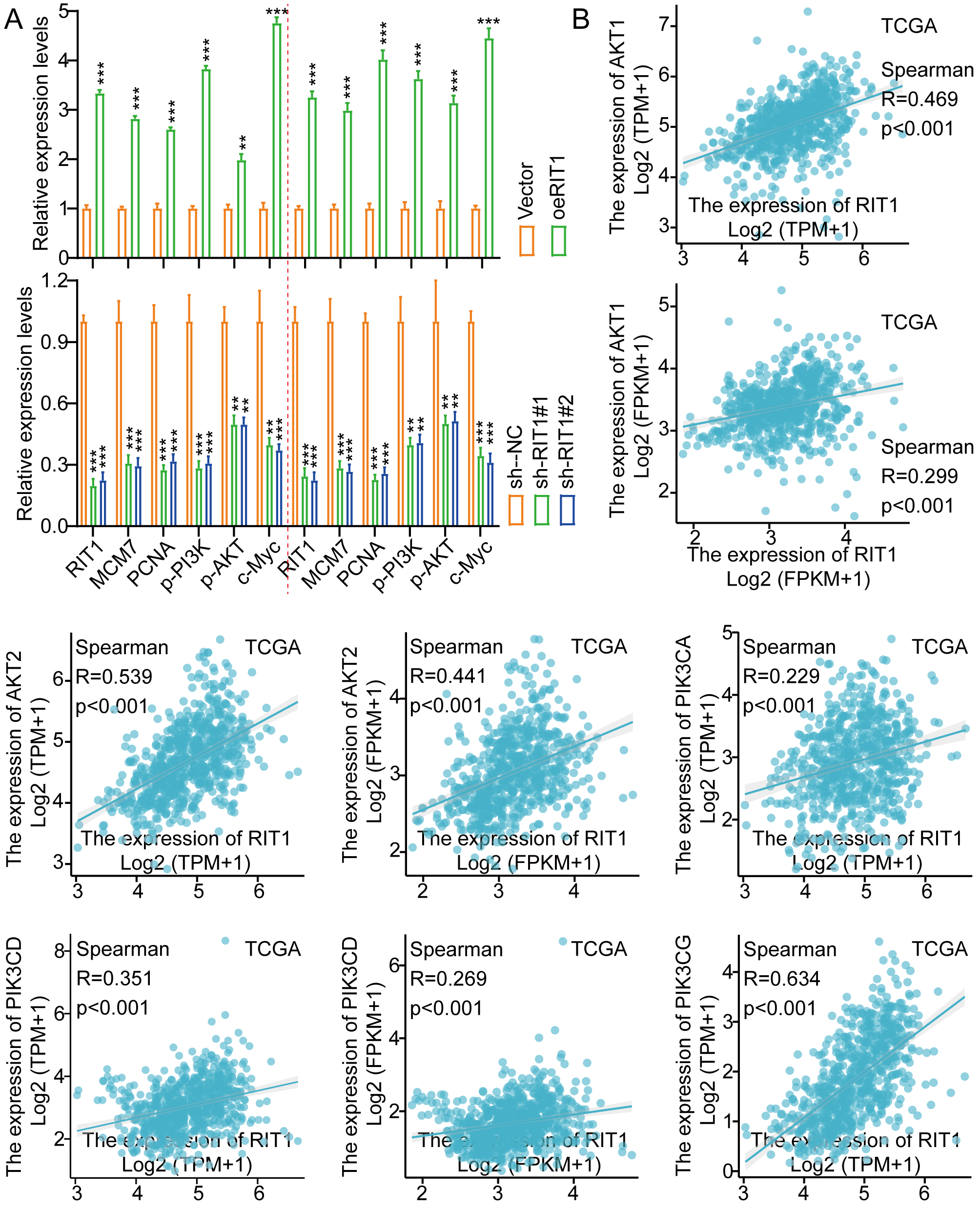


**Supplementary Figure 4: RIT1 is positively correlated with molecules related to the PI3K/AKT signaling pathway.**

**A.** Statistical bar graph of the relative expression levels of RIT1, MCM7, PCNA, p-PI3K, p-AKT and c-Myc after overexpression and knockdown of RIT1. **B.** Analysis of the TCGA database showed that RIT1 was positively correlated with molecules related to the PI3K/AKT signaling pathway. Statistical significance was tested using one-way ANOVA (Dunnett’s tests) for several comparisons and two-tailed t-tests. ***P* < 0.01 and ****P* < 0.001.


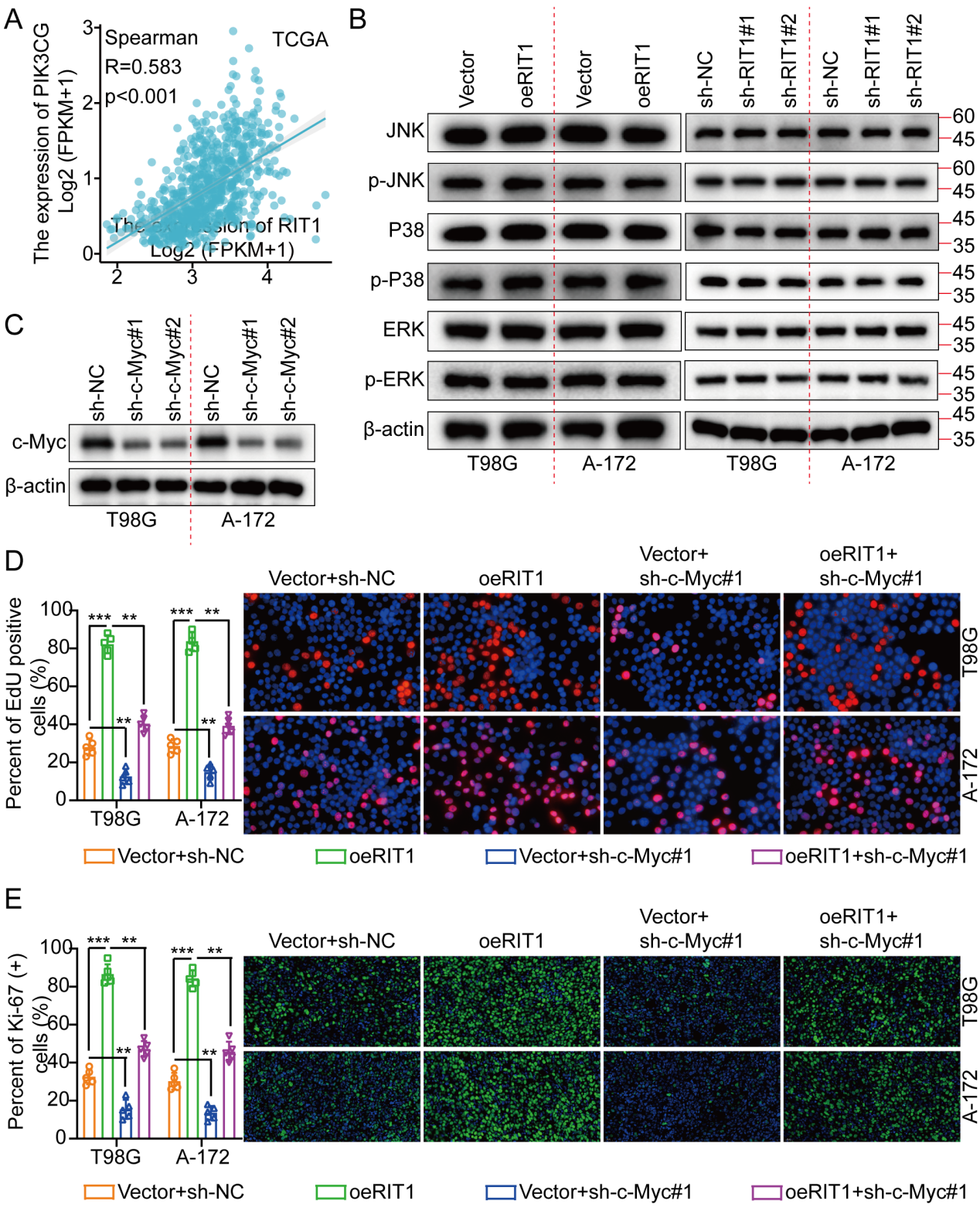


**Supplementary Figure 5: RIT1 promotes glioma proliferation through c-Myc.**

**A.** Analysis of the TCGA database showed that RIT1 was positively correlated with molecules related to the PI3K/AKT signaling pathway. **B.** The expression levels of JNK, p-JNK, P38, p-P38, ERK and p-ERK was analyzed by WB in Vector, oeRIT1, sh-NC, sh-RIT1#1 and sh-RIT1#2 groups. **C.** Validation of c-Myc knockdown efficiency via WB. **D.** Cell growth was evaluated using EdU assay and histogram analysis across different treatment groups. **E.** Representative pictures of Ki-67 staining at different treatment groups. Data are presented as Mean ± s.d from five independent experiments. Statistical significance was tested using one-way ANOVA (Dunnett’s tests) for several comparisons and two-tailed t-tests. ***P* < 0.01 and ****P* < 0.001.


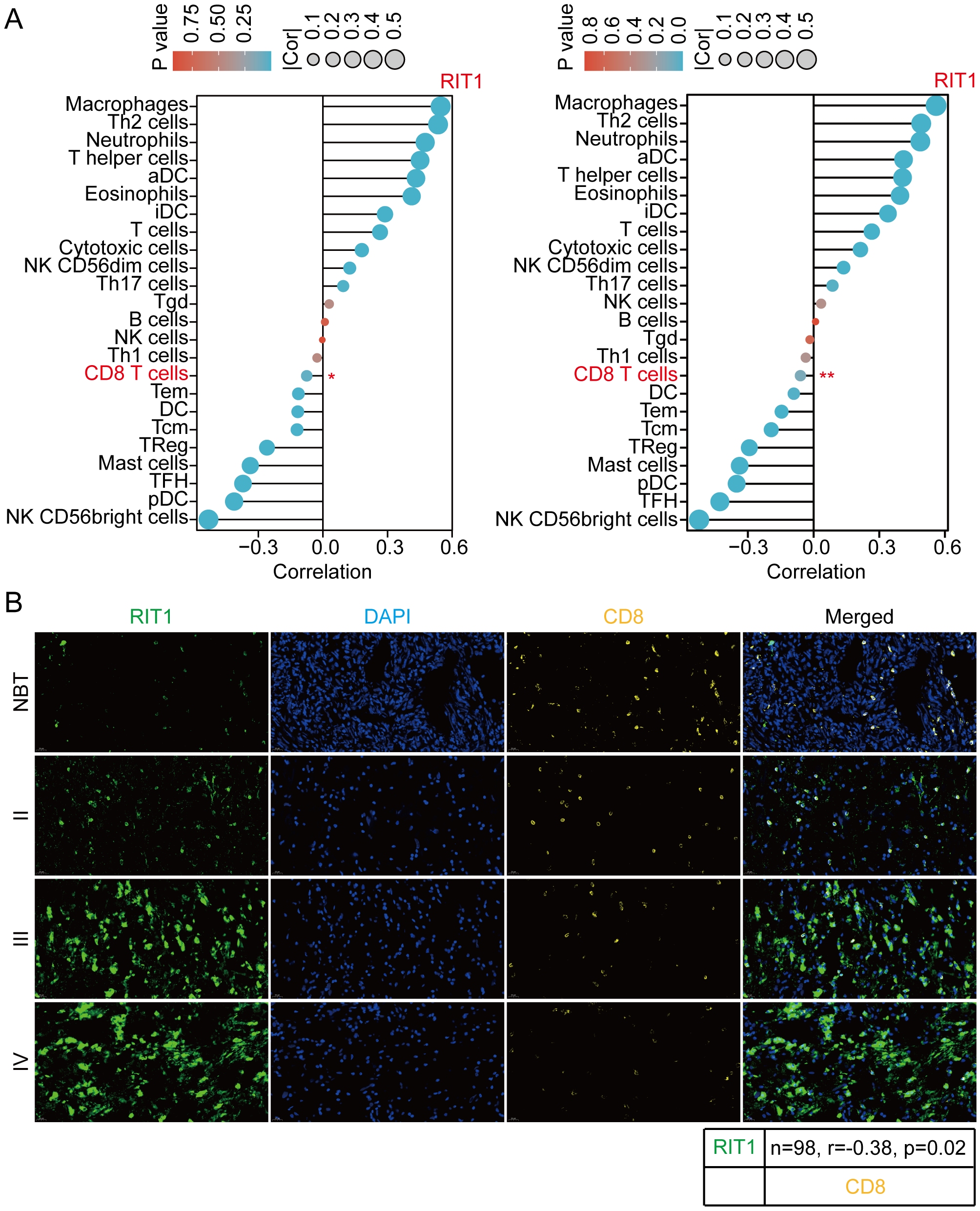


**Supplementary Figure 6: RIT1 is negatively correlated with CD8 positive T cells.**

**A.** TCGA database analysis showed that RIT1 was negatively correlated with CD8+ T cells. **B.** TCGA database analysis showed that RIT1 was negatively correlated with CD8+ T cells


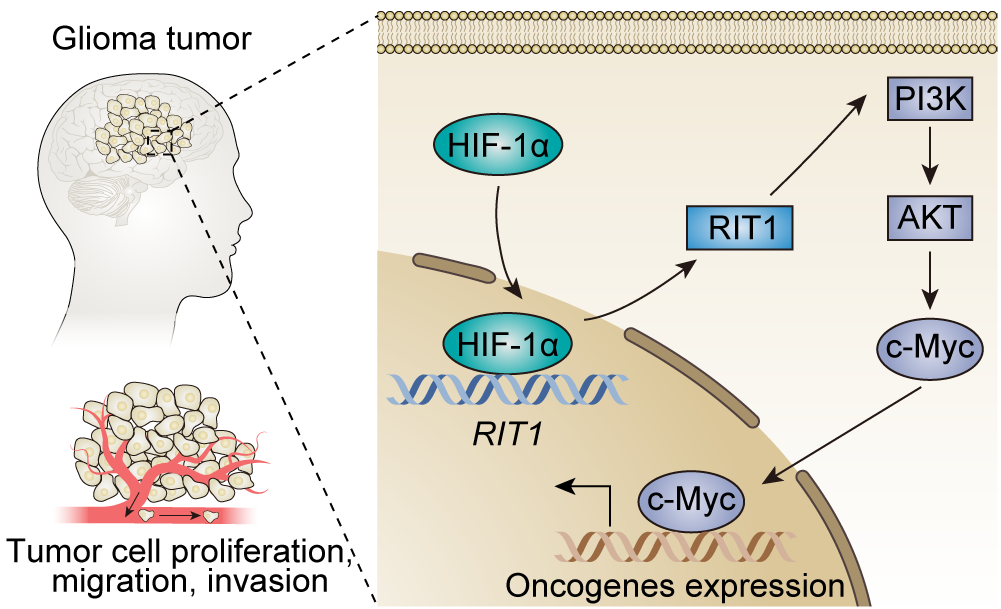


**Supplementary Figure 7: Schematic diagram of the mechanism.** RIT1 is highly expressed in gliomas, and promotes tumor progression by inhibiting apoptosis and promoting cell cycle progression. Mechanistically, RIT1 downstream promotes glioma growth by activating PI3K/AKT/c-Myc, and RIT1 upstream is mediated by HIF-1α, which promotes the transcriptional activity and expression level of RIT1.
